# The Role of Clonal Evolution on Progression, Blood Parameters, and Response to Therapy in Multiple Myeloma

**DOI:** 10.3389/fonc.2022.919278

**Published:** 2022-07-19

**Authors:** Sarah Sandmann, Katharina Karsch, Peter Bartel, Rita Exeler, Tobias J. Brix, Elias K. Mai, Julian Varghese, Georg Lenz, Cyrus Khandanpour

**Affiliations:** ^1^ Institute of Medical Informatics, University of Münster, Münster, Germany; ^2^ Department of Medicine A, Hematology, Oncology and Pneumology, University Hospital Münster, Münster, Germany; ^3^ Institute of Human Genetics, University Hospital Münster, Münster, Germany; ^4^ Department of Internal Medicine V, Heidelberg University Hospital, Heidelberg, Germany; ^5^ University Medical Center Schleswig-Holstein Campus Lübeck, University of Lübeck, Lübeck, Germany

**Keywords:** multiple myeloma, clonal evolution, survival, prognosis, chromosomal alteration

## Abstract

**Introduction:**

A variety of biomarkers are considered for diagnosis (e.g., β2-microgobulin, albumin, or LDH) and prognosis [e.g., cytogenetic aberrations detected by fluorescence *in situ* hybridization (FISH)] of multiple myeloma (MM). More recently, clonal evolution has been established as key. Little is known on the clinical implications of clonal evolution.

**Methods:**

We performed in-depth analyses of 25 patients with newly diagnosed MM with respect to detailed clinical information analyzing blood samples collected at several time points during follow-up (median follow-up: 3.26 years since first diagnosis). We split our cohort into two subgroups: with and without new FISH clones developing in the course of disease.

**Results:**

Each subgroup showed a characteristic chromosomal profile. Forty-three percent of patients had evidence of appearing new clones. The patients with new clones showed an increased number of translocations affecting chromosomes 14 (78% vs. 33%; *p* = 0.0805) and 11, and alterations in chromosome 4 (amplifications and translocations). New clones, on the contrary, were characterized by alterations affecting chromosome 17. Subsequent to the development of the new clone, 6 out of 9 patients experienced disease progression compared to 3 out of 12 for patients without new clones. Duration of the therapy applied for the longest time was significantly shorter within the group of patients developing new clones (median: 273 vs. 406.5 days; *p* = 0.0465).

**Discussion:**

We demonstrated that the development of new clones, carrying large-scale alterations, was associated with inferior disease course and shorter response to therapy, possibly affecting progression-free survival and overall survival as well. Further studies evaluating larger cohorts are necessary for the validation of our results.

## 1 Introduction

Multiple myeloma (MM) is a hematologic malignancy characterized by monoclonal expansion of plasma cells in the bone marrow (1). Clinical presentation is very heterogeneous, ranging from a rather indolent course to clinically aggressive plasma cell leukemia.

The diagnostic criteria of MM have expanded in recent years and now include biomarkers for disease malignancy such as bone marrow plasma cell count, free light chain ratio, and number of focal lesions on MRI, in addition to the well-established CRAB criteria (2).

The prognostic significance of cytogenetic aberrations is well described; t(4;14), t(14;16), and del(17p) are included in Revised International Staging System (R-ISS) risk stratification (3, 4). A change in the mutational profile or clonal composition between two or more time points is referred to as clonal evolution (5, 6). Models describing clonal evolution can be categorized as linear, branched, punctuated, or neutral (7).

The molecular basics of clonal evolution in MM have been studied and reviewed in depth (5, 8–10). It has been observed that the complexity of the MM tumor genome increased over time (11) and that cytogenetic heterogeneity is of prognostic significance in newly diagnosed MM patients treated with bortezomib (12). However, more information on clinical implications of clonal evolution is needed.

We hypothesize that patients with MM, characterized by new clones emerging after first diagnosis, show poor prognosis compared to patients without new clones. We define a new clone as a gain of aberrations and, thus, increased heterogeneity of a tumor. The gain can affect both healthy cells, leading to branched clonal evolution with independent clones, or clones already present at first diagnosis, leading to a derivative of these clones. To explore our hypothesis, we analyzed a set of 25 patients with MM, split into two subgroups: with or without new clones. For these patients, we correlated clinical data on progression, chromosomal profiles, blood parameters, and therapies, based on blood samples collected at several time points during follow-up.

## 2 Methods

### 2.1 Study Population

A cohort of 25 patients with MM and comparable therapies applied, treated at the University Hospital Münster, was analyzed. All data were collected and analyzed in accordance with relevant ethical guidelines and principles of the Declaration of Helsinki. The ethical review boards of the University of Münster and the Ärztekammer Westfalen-Lippe approved this study (2018-452-f-S). All patients gave their written informed consent. Detailed information on every patient is provided in **Supplementary Table 1**; a summary of the study population’s main characteristics is provided in **Supplementary Table 2**.

Data were collected between December 2014 and March 2021. All patients were monitored starting with first diagnosis of MM (exceptions: for UPN09, information on laboratory parameters and therapies applied was available at diagnosis of a plasmocytoma, 2 years prior to diagnosis of MM; for UPN16, information on laboratory parameters was available 4 months prior to first diagnosis of MM). Median time of follow-up after first diagnosis of MM was 3.62 years (IQR = 2.01–5.00 years). Forty percent of patients were female. At first diagnosis, patients were in median 58 years old (IQR = 53–64 years). Nine out of 25 patients received autologous stem cell transplantation (SCT). Five overall survival (OS) events occurred during follow-up (see *Section 2.4 Statistical Methods* for the definition of OS). Since this was the primary endpoint of the study, data on cytogenetic aberrations were available for all patients at several time points. Analyses on blood samples of the patients were performed between 2014 and 2021 at the Institute of Human Genetics Münster. A median of 3 samples per patient was analyzed during follow-up (range: 1–11 analyses during follow-up). Additionally, information on therapy and laboratory parameters (κ and λ light chains measured in serum), lactate dehydrogenase (LDH), and gradient of monoclonal protein (M-gradient) was available for every patient at several time points during follow-up. A visual summary of available data is provided in **Supplementary Figure 1**, detailed information on laboratory parameters is provided in **Supplementary Figures  2**–**4**.

Based on clonal evolution, we split up our cohort into two subgroups: For subgroup 1, no new clones were detected in the course of disease (patients UPN05 to 16) based on cytogenetic findings using the fluorescence *in situ* hybridization (FISH) approach. All clones were already present at first analysis with FISH. For subgroup 2, at least one completely new clone was developed in the course of the disease, which was not detected at any previous time point (patients UPN17 to 25); i.e., a gain of aberrations occurred. Four patients (UPN01 to 04) were excluded from subgroup analysis, because they had no follow-up FISH samples available.

### 2.2 Fluorescence *In Situ* Hybridization Analysis

FISH was performed as described previously (13, 14). Briefly, CD138-purified plasma cells were analyzed. The following commercial available probes from Cytocell (Cytocell, Cambridge, UK), MetaSystems (MetaSystems Probe GmbH, Altlußheim, Germany), CytoTest (CytoTest, Rockville, USA), and Abbott Molecular (Abbott, Green Oaks, USA) were used: Cytocell CKS1B/CDKN2C (P18) Amplification/Deletion Probe (1p32.3, 1q21); Cytocell D13S319 Plus Deletion Probe (13q14.2, 13qter); MetaSystems XL TP53/NF1 Deletion Probe (17p13.1, 17q11.2); MetaSystems XL Iso(17q) Deletion Probe (17p13, 17q22); MetaSystems XL MYC BA Break Apart Probe (8q24); MetaSystems XL E2A Break Apart Probe (19q13); CytoTest NSD1/TERT FISH Probe Kit (5q35, 5p15); Abbott Molecular LSI ATM/CEP11 11 FISH Probe (11cen, 11q22); Cytocell probes for centromeric regions (D3Z1, D7Z1, D9Z3, D15Z4); Cytocell IGH/FGFR3 Plus Translocation, Dual Fusion (4p16.3, 14q32.33); Cytocell IGH/MAF Translocation, Dual Fusion (14q32, 16q23); Cytocell IGH/MAFB Translocation, Dual Fusion (14q32, 20q12); Cytocell IGH/MYEOV Translocation, Dual Fusion (11q13, 14q32); and Cytocell IGH Plus Breakapart Probe (14q32.33). At least 100 interphase nuclei were analyzed for each probe (exception: UPN15 at time point 1: 81 nuclei, time point 2: 56 nuclei). Cutoff levels for all probes were ~5%. Read out was done by human genetics experts in Münster.

### 2.3 Clonal Evolution

For every patient, data on present and absent chromosomal aberrations were available for 1 to 11 time points. As alterations were determined using FISH, the number of cells affected by alteration divided by the total number of evaluated cells corresponds to the cancer cell fraction (CCF). Clonal evolution was manually reconstructed based on CCF (15). To reconstruct clonal evolution also in the presence of a few time points, we applied an approach estimating the clonal development between measured time points (16). Detailed information on detected alterations, CCFs, and assigned clusters is available in **Supplementary Table 3**.

### 2.4 Statistical Methods

Plots visualizing clonal evolution were generated using R 4.1.2 (17) and R package “fishplot” (18). Complex plots were developed, combining fishplots with diagrams visualizing therapy information and laboratory parameters over time. In case clonal evolution could not be reconstructed uniquely based on available data, all possible versions were reported. Plots for every patient are available as **Supplementary Figures 5**–**29**.

Analysis of progression-free survival (PFS) and OS was performed using R 4.1.2 (17). For PFS, we analyzed the time between first diagnosis and first progression. Additionally, we considered the time between last FISH (subgroup 1)/FISH detecting the new clone (subgroup 2) and next progression. For OS, we analyzed the time between first diagnosis and death of a patient. Kaplan–Meier curves were calculated using R 4.1.2 (17) and R packages “survival” (19) and “survminer” (20).

## 3 Results

In this study, we analyzed clonal evolution in patients with MM, aiming at identifying differences with respect to disease progression, distinct chromosomal profiles, laboratory parameters, and response to therapy.

### 3.1 Disease Progression

**Figure 1:**
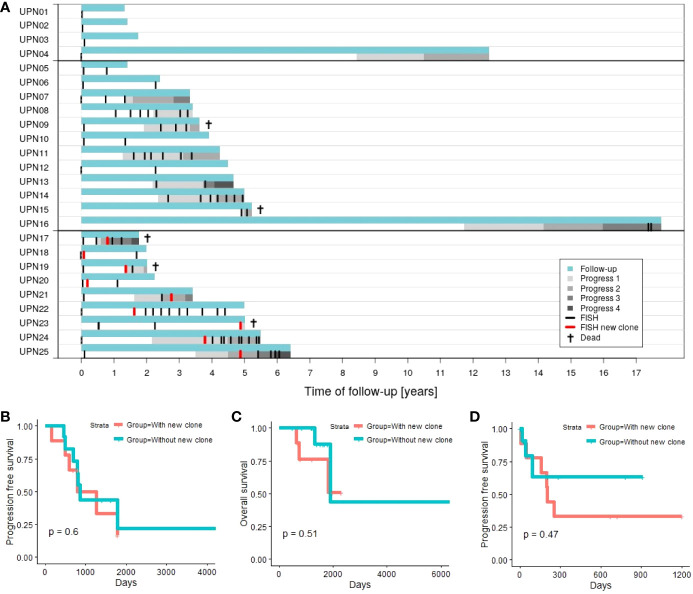
**(A)** Time of follow-up for patients with 1 time point of aberration analysis (patients UPN01 to 04), patients with >1 time point of aberration analysis and no new clone emerging in the course of disease (patients UPN05 to 16; subgroup 1), and patients with >1 time point of aberration analysis and a new clone emerging in the course of disease (patients UPN17 to 25; subgroup 2). **(B)** Progression-free survival (first progression after first diagnosis) comparing subgroup 1 vs. subgroup 2. **(C)** Overall survival comparing subgroup 1 vs. subgroup 2. **(D)** Progression-free survival in relation to chromosomal alterations comparing subgroup 1 (next progression subsequent to last FISH) vs. subgroup 2 (next progression subsequent to detection of the new clone).

**Figure 1A** provides an overview of follow-up, time points of performed FISH, and progression data.

For 8 out of 12 patients (67%) in subgroup 1 (without new clone), disease progression could be observed. Similarly, 6 out of 9 patients (67%) in subgroup 2 (with new clone) experienced progression in the course of follow-up. Investigating whether disease progression takes place earlier in subgroup 2, we analyzed PFS (time between first diagnosis and first progression). However, no significant results could be obtained (**Figure 1B**; *p* **= 0.6**).

Two out of 12 patients (17%) in subgroup 1 died within our observation period. In contrast, 3 out of 9 patients (33%) deceased in subgroup 2. Analysis of OS (time between first diagnosis and death of a patient) did not indicate significant differences between both subgroups (**Figure 1C**; *p* **= 0.51**).

Additionally, we consider PFS in relation to analysis of chromosomal alterations. The time span between last FISH (subgroup 1) or detection of the new clone (subgroup 2), and the next subsequent progression (**Figure 1D**) is evaluated. Four out of 12 patients in subgroup 1 (patients UPN05, 06, 10, and 12) and 3 out of 9 in subgroup 2 (patients UPN18, 20, and 22) never experienced any progression within time of follow-up. Additionally, 5 out of the remaining 8 patients in subgroup 1 did not experience any further disease progression subsequent to last FISH. Analysis of PFS did not reveal any significant differences between both subgroups (*p* **= 0.45**). Subsequent to the development of the new clone, 6 out of 9 patients experienced disease progression compared to 3 out of 12 for patients without new clones.

### 3.2 Chromosomal Profiles

Patients with MM can be characterized by specific chromosomal profiles: Translocations involving chromosome 14 [especially t(4;14)], gains and amplifications affecting chromosomal region 1q21, as well as deletion of 17p are indicators for adverse prognosis (3, 4, 9, 10, 12).

**Figure 2A** visualizes the chromosomal profiles of all patients considered in this study for the whole time of follow-up (for detailed information on cytogenetic aberrations detected by FISH, see **Supplementary Table 3**; for detailed information on clonal evolution of each patient, see **Supplementary Figures 5**–**29**). As expected, a majority of patients featured translocations affecting chromosome 14 (12 out of 25; 48%), amplifications affecting chromosome 1 (14 out of 25; 56%), and/or deletions affecting chromosome 17 (9 out of 25; 36%). Additionally, 14 out of 25 patients (56%) showed deletions in chromosome 13. For 10 out of 25 patients (40%), alterations affecting chromosome 11 (translocations and amplifications) were detected.

**Figure 2:**
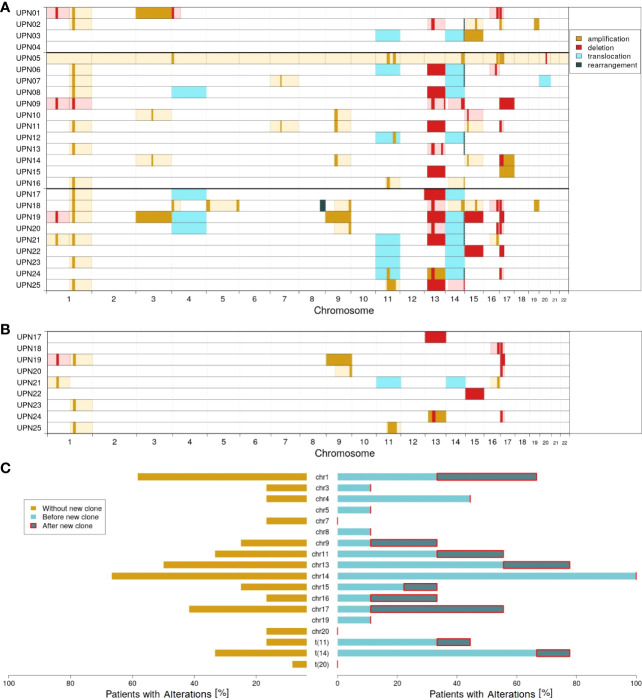
Molecular characterization of the cohort; amplifications (dark yellow), deletions (red), translocations (light blue), and rearrangements (dark green) according to FISH probes. Light colors (yellow and red) indicate likely interpretation of the observed FISH results. **(A)** Overview of all alterations detected. **(B)** Alterations characterizing the new clones observed in patients UPN17 to 25. **(C)** Proportion of patients with altered chromosomes: without the new clone (dark yellow), with the new clone before (light blue), and after (dark blue) detection of the new clone.

Comparing subgroups 1 and 2, considerable differences could be observed. While only 33% of all patients in subgroup 1 (4 out of 12) were characterized by translocation of chromosome 14, it was 78% in subgroup 2 (7 out of 9; *p* = 0.0805). With respect to all alterations affecting chromosome 14, a relation of 67% vs. 100% could be observed (8 out of 12 vs. 9 out of 9; *p* = 0.1038). Further major differences affected translocation of chromosome 11 (17% vs. 44%; 2 out of 12 vs. 4 out of 9) and changes in chromosome 4 (17% vs. 44%; 2 out of 12 vs. 4 out of 9).

Within subgroup 2, further differences could be observed comparing alterations detected by the first FISH and alterations characterizing the newly developed clones. **Figure 2B** visualizes all cytogenetic aberrations characterizing the new clones. In **Figure 2C**, a summary on chromosome level is provided.

It appeared striking that only 1 out of 9 patients in subgroup 2 had an initial alteration in chromosome 17. The ratio increased to 5 out of 9 when considering newly developed clones. Similarly, 4 out of 9 patients acquired alterations in chromosome 1. By contrast, only 1 out of 9 patients (UPN21) acquired an additional alteration in chromosome 14 in the course of clonal evolution. However, this patient already had a rearrangement in chromosome 14 at first diagnosis. Furthermore, no new alterations are acquired in chromosomes 4 (alterations detected at first diagnosis in 44%; 4 out of 9), 3, 5, 8, and 18 (11% each; 1 out of 9).

### 3.3 Laboratory Parameters

**Figure 3:**
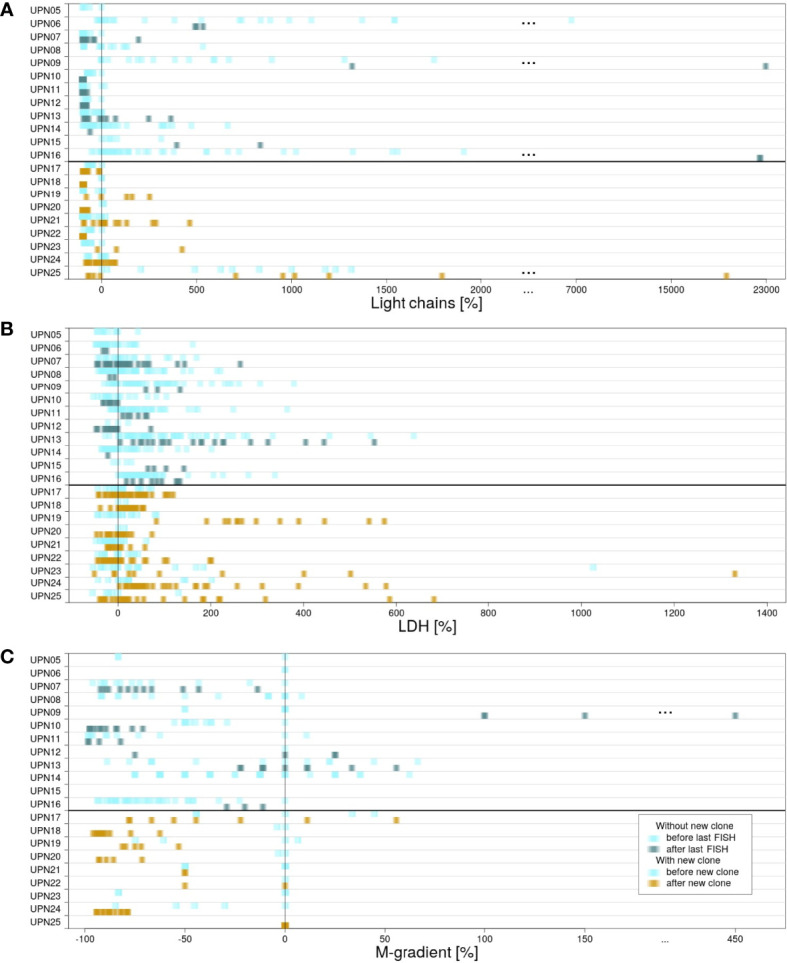
Differences in measured laboratory parameters. For patients in subgroup 1, laboratory parameters measured before (light blue) and after (blue/green) last FISH. For patients in subgroup 2, laboratory parameters measured before (light blue) and after (yellow) detection of the new clone. **(A)** κ and λ light chains measured in serum. **(B)** LDH activity. **(C)** Gradient of monoclonal protein (M-gradient).

For all patients, information on the presence of κ and λ light chains in serum, increased LDH activity, and gradient of monoclonal protein (M-gradient) was available, measured at several time points in the course of disease. **Figure 3** visualizes the development of laboratory parameters in the two subgroups. Color indicates the time point at which measurements were conducted. For patients without new clones, we compared values measured prior to and after last FISH. For patients with new clones, we compared values measured prior to and after the development of the new clone.

For κ and λ light chains, no correlation between the values measured and the time point at which they were measured could be observed. This observation is true for both subgroups (for details, see **Supplementary Figure 2**).

LDH serum levels measured in subgroup 1 were heterogeneously distributed. The highest as well as the lowest values were scattered over the time of follow-up, independent of therapy. In contrast, correlation with respect to time point could be observed for subgroup 2. In 8 out of 9 cases, the highest values of LDH activity were measured subsequent to the development of the new clones. However, detailed evaluation of the results revealed that values did not show a continuous increase. Instead, peaks were usually observed within 1 year after development of the new clone. Subsequently, LDH activity decreased again until the end of follow-up (**Supplementary Figure 3**). Significant correlation to therapies applied could not be observed.

For M-gradient, no certain pattern was associated prior to the development of myeloma or upon the development of a new clone (for details, see **Supplementary Figure 4**). Patient UPN09 (subgroup 1) is an exception from this observation. A significant increase in M-gradient can be observed (*p*_UPN09_ = 0.0065). The patient died within less than 4 years after the first diagnosis.

### 3.4 Response to Therapy

For treatment of MM, a variety of therapies are available. In the course of disease progression, time to therapy failure is known to decrease (21).

**Figure 4** provides an overview of therapies applied in subgroups 1 and 2 (for detailed information on therapy for every patient, see **Supplementary Figures 5**–**29**). In general, patients in both subgroups received comparable therapies. However, while patients in subgroup 1 were characterized by many therapies being applied for a long time (**Figure 4A**), switches in therapy after a short time could be observed for subgroup 2 (**Figure 4B**). While all patients received at least one therapy for ≥100 days, 83% (10 out of 12) received at least one therapy for ≥200 days in subgroup 1 vs. 55% (5 out of 9) in subgroup 2 (75% vs. 33% for therapies received for ≥300 days).

**Figure 4:**
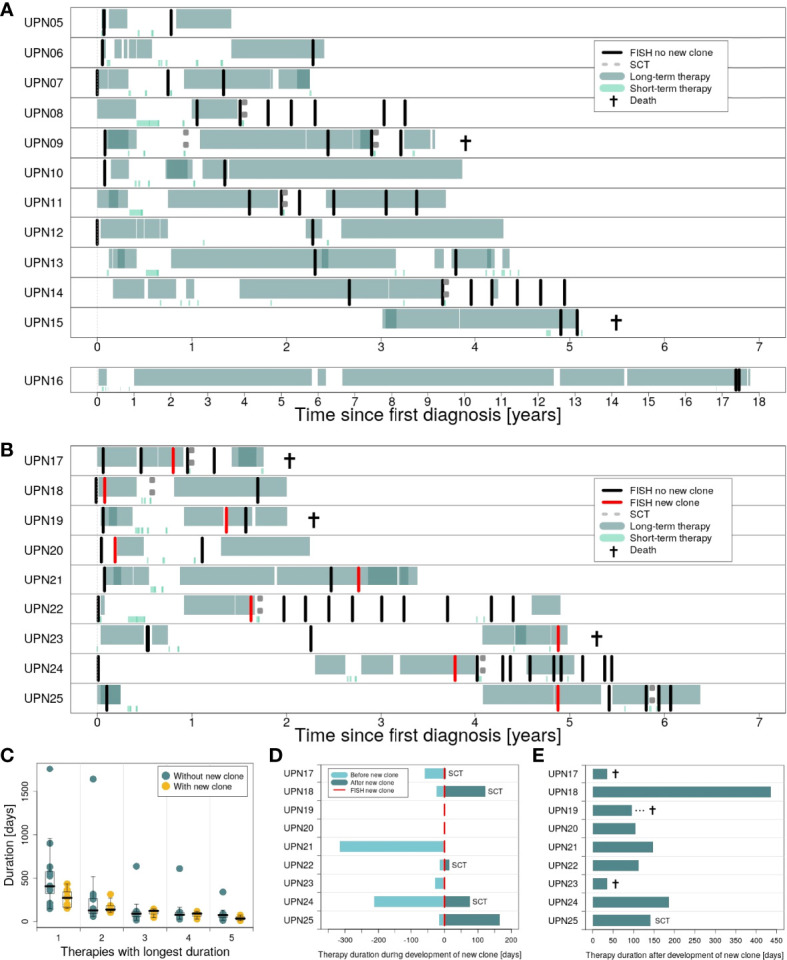
Duration of therapies applied in the course of disease for patients in subgroups 1 and 2. **(A)** Long-term vs. short-term therapies for patients in subgroup 1 (patients UPN05 to 16). **(B)** Long-term vs. short-term therapies for patients in subgroup 2 (patients UPN17 to 25). **(C)** Therapies with the longest duration comparing subgroup 1 vs. subgroup 2. **(D)** Duration of therapies for subgroup 2, applied during development of the new clone. **(E)** Duration of the first therapy applied after development of the new clone.

We compared both subgroups with respect to the top 5 therapies applied for the longest time (**Figure 4C**). Only marginal differences could be observed for therapies 2 to 5. However, for the therapy applied for the longest time, subgroups showed major differences. Analysis of our data by Mann–Whitney *U* test revealed a significant difference between both subgroups: For patients with new clones, duration of the longest therapeutic regimes was significantly shorter compared to patients without new clones (*p* = 0.0465; median subgroup 1: 406.5 days, range: 150–1,764 days; median subgroup 2: 273 days, range: 152–434 days).

Detailed information on the duration of therapies applied during and after development of the new clones for patients in subgroup 2 is provided in **Figures 4D, E**. For two patients, UPN19 and 20, the new clone was developed in between two therapeutic regimes. Five patients in subgroup 2 received SCT, with four out of 5 right after the development of the new clone. Immediate change in therapy after the development of the new clone was observed for 3 patients (UPN17, 21, and 23), of whom two died within less than 50 days.

## 4 Discussion

Clonal evolution and—as a result—increasing clonal heterogeneity have been studied widely in MM (22–27). It can be observed not only during the disease course as an adaptation to different treatments, but also at the same time point of the disease stage with different clones being present at different locations of the body (e.g., in focal lesions and the iliac crest; 28). Even within a compartment, such as the bone marrow, the expression pattern of plasma cells can differ depending on their position (29). Adding further complexity is the fact that the patient’s treatment course can be especially heterogeneous regarding the sequence of therapeutic regimes the patient received. Finally, clonal evolution can be observed on a rather large scale such as chromosomal alterations or smaller scale with mutations or small range deletions or amplifications.

In all cases, clonal evolution contributes to the fact that the disease course of MM is characterized by a shorter PFS from one line of therapy to the other (21). It remains to be defined how different clonal branches and different sites of the body at different time points might be best detected, classified, and accordingly treated. Yet, in clinical routine, performing whole-genome sequencing or biopsies from different sites is not feasible right now. Additionally, identifying these sites, e.g., by PET-CT or MRI, is equally difficult.

In this study, we performed an in-depth analysis of patients with MM. Our analyses focused on clinical differences in patients with and without new clones developing in the course of disease. Clones were characterized by larger-scale alterations, which could be detected by FISH—a method very widely established and rather affordable in an outpatient setting. Integration of data from a variety of sources—disease progression, chromosomal profiles, laboratory parameters, and applied therapies—provided us with the unique option to study the interplay of diverse features characterizing patients with MM. The limiting factors are that we focused on large-scale alterations, clones might have been missed due to limited sensitivity of FISH (30), and the disease course and treatment sequences are quite heterogeneous. Additionally, our patient cohort is rather small. Dividing our cohort into two subgroups—with and without new clones—further limits the statistical power of our results.

With these limitations in mind, we could make the following observations: In our cohort, new large-scale chromosomal aberrations developed quite frequently (9/21 patients—43%). Clones emerging with chromosomal aberrations frequently featured alteration of chromosomes 1 and 17 (44% each; 77% in total). Both chromosomes have been described to play a role in myeloma progression, with del(17p) affecting the function of *TP53* with its known negative factor influencing the course of hematological malignancies (31). In addition, chromosome 1 alterations have been shown to be associated with an inferior disease course leading to the amplification of genes such as *BCL9*, *MCL1*, *CKS1B*, and *ANP32E*. These genes are either implicated in the inhibition of apoptosis, enhancement of cell cycle, or epigenetic modification (9, 10, 12, 25, 32–34).

With all the limitations due to the small size of our cohort and thus limited statistical power, emergence of a large-scale genomic alteration negatively influenced length of response of therapy and potentially PFS and OS, although both did not reach significance. It also led to an overall shorter PFS to each line subsequent to the emergence of a new clone. This observation is in line with previously reported results on clonal evolution negatively influencing prognosis (35). We evaluated whether emergence of a new clone could be correlated with laboratory findings. LDH activity reached higher peaks after clonal evolution, although the specific courses were quite heterogeneous, reflecting that the disease might initially respond to therapy, but then again relapse.

Altogether, we can show that large-scale genomic clonal evolution is associated with an inferior disease course with shorter response duration to subsequent new lines, possibly shorter PFS and OS. It affects, at a high degree, chromosomes 1 and 17, which confer a negative prognosis already at initial diagnosis. These results warrant additional studies with larger groups treated more homogeneously for confirmation. For these groups, collection of detailed information on progression, blood parameters, and response to therapy during follow-up is required in addition to analysis of genomic alterations. The number of patients analyzed is a limitation of our study. Our goal was to use an approach to follow clonal evolution, which is widely used in a clinical setting. We screened, in our database, more than 600 patients and only found these patients, for whom we have consecutive FISH data combined with a variety of clinical data. The strength of our study is that we can connect clonal status with standard laboratory parameters and clinical outcome. Most clinical trials focus on the specific setting with follow-up to the next line of treatment, but long-term data over the course of different courses are not easily available. What we show is “real-world data” regarding frequency of large-scale genomic alterations and that clonal evolution is, as not surprising, really heterogeneous with regard to its association with different clinical parameters. Yet, the search for clonal large-scale genomic alterations can actually be achieved also in an outpatient setting. It would then require discussion with the patient, in which additional therapeutic steps might be required to counteract the effect of such variation.

## Data Availability Statement

Original data presented in the study are included in the article/**Supplementary Material**. Further inquiries can be directed to the corresponding authors.

## Ethics Statement

The study was reviewed and approved by ethical review boards of the University of Münster and the Ärztekammer Westfalen-Lippe (2018-452-f-S). The patients provided their written informed consent to participate in this study.

## Author Contributions

KK, RE, TJB, and CK collected patient samples. SS and KK performed data analyses. All authors interpreted and discussed the data. SS, KK, PB, CK, and EKM wrote the manuscript. CK, JV, and GL coordinated and supervised the project. All authors read, revised, and approved the final version of the manuscript.

## Funding

The work was supported by the Jose Carreras Leukämie Foundation (DJCLS 17R/2018), partially by the Deutsche Krebshilfe (70112392), Deutsche Forschungsgemeinschaft (KH331/2-3), the intramural funding of the faculty of Medicine at University Hospital of Muenster (Kha2/002/20), and a grant from Bundesministerium für Bildung und Forschung (HiGHmed 01ZZ1802V).

## Conflict of Interest

EKM reports consulting or Advisory Role, Honoraria, Research Funding, and Travel Accommodations and Expenses from Bristol Myers Squibb/Celgene, GlaxoSmithKline, Janssen Cilag, Sanofi and Takeda.

Remaining authors declare that the research was conducted in the absence of any commercial or financial relationships that could be construed as a potential conflict of interest.

## Publisher’s Note

All claims expressed in this article are solely those of the authors and do not necessarily represent those of their affiliated organizations, or those of the publisher, the editors and the reviewers. Any product that may be evaluated in this article, or claim that may be made by its manufacturer, is not guaranteed or endorsed by the publisher.

## References

[1] Palumbo A Anderson K. Multiple Myeloma. N Engl J Med (2011) 364:1046–60. doi: 10.1056/204NEJMra1011442205 21410373

[2] Rajkumar SV Dimopoulos MA Palumbo A Blade J Merlini G Mateos M-V. International Myeloma Working Group Updated Criteria for the Diagnosis of Multiple Myeloma. Lancet Oncol (2014) 15:e538–48. doi: 10.1016/S1470-2045(14)70442-5215 25439696

[3] Palumbo A Avet-Loiseau H Oliva S Lokhorst HM Goldschmidt H Richardson LR. Revised International Staging System for Multiple Myeloma: A Report From International Myeloma Working Group. J Clin Oncol (2015) 33:2863–9. doi: 10.1200/JCO.2015.61.2267208 PMC4846284 26240224

[4] Rajkumar SV. Multiple Myeloma: 2020 Update on Diagnosis, Risk-Stratification and Management. Am J Hematol (2020) 95:548–67. doi: 10.1002/ajh.25791212 32212178

[5] Bolli N Avet-Loiseau H Wedge DC Van Loo P Alexandrov LB Martincorena I. Heterogeneity of Genomic Evolution and Mutational Profiles in Multiple Myeloma. Nat Commun (2014) 5:496–511. doi: 10.1038/ncomms3997 PMC3905727 24429703

[6] Furukawa Y Kikuchi J. Molecular Basis of Clonal Evolution in Multiple Myeloma. Int J Hematol (2020) 111:496–511. doi: 10.1007/s12185-020-02829-6199 32026210

[7] Davis A Gao R Navin N. Tumor Evolution: Linear, Branching, Neutral or Punctuated? Biochim Biophys Acta Rev Cancer (2017) 1867::496–511. doi: 10.1016/j.bbcan.2017.01.003197 PMC5558210 28110020

[8] Castaneda O Baz R. Multiple Myeloma Genomics - A Concise Review. Acta Med Acad (2019) 48:57–67. doi: 10.5644/ama2006-124.242192 31264433

[9] Merz M Jauch A Hielscher T Mai EK Seckinger A Hose D. Longitudinal Fluorescence *in Situ* Hybridization Reveals Cytogenetic Evolution in Myeloma Relapsing After Autologous Transplantation. Haematologica (2017) 102:1432–8. doi: 10.3324/haematol.2017.168005 PMC5541876 28495913

[10] Merz M Hielscher T Schult D Mai EK Raab MS Hillengass J. Cytogenetic Subclone Formation and Evolution in Progressive Smoldering Multiple Myeloma. Leukemia (2019) 34:1192–6. doi: 10.1038/s41375-019-0634-2 PMC7214236 31712777

[11] Keats JJ Chesi M Egan JB Garbitt VM Palmer SE Braggio E. Clonal Competition With Alternating Dominance in Multiple Myeloma. Blood (2012) 120:1067–76. doi: 10.1182/blood-2012-01-405985 PMC3412330 22498740

[12] Merz M Jauch A Hielscher T Bochtler T Schönland SO Seckinger A. Prognostic Significance of Cytogenetic Heterogeneity in Patients With Newly Diagnosed Multiple Myeloma. Blood Adv (2017) 2:1–9. doi: 10.1182/bloodadvances.2017013334 29344579 PMC5761630

[13] Bayani J Squire JA. Fluorescence *in Situ* Hybridization (FISH). Curr Protoc Cell Biol (2004) 22:22.4.1–52. doi: 10.1002/0471143030.cb2204s23187 18228455

[14] Brune T Riepe FG Beier K Exeler R Louwen F Garritsen H. Differentiation Of188single Populations in a Bidirectional Mixed Lymphocyte Culture Using X and Y Chromosome-Specific FiSH Markers. J Immunol Methods (2002) 266:105–10. doi: 10.1016/s0022-1759(02)00107-2190 12133626

[15] da Silva-Coelho P Kroeze LI Yoshida K Koorenhof-Scheele TN Knops R van de Locht LT. Clonal Evolution in Myelodysplastic Syndromes. Nat Commun (2017) 8:639–43. doi: 10.1038/194ncomms15099. 193. PMC5530598 28429724

[16] Reutter K Sandmann S Rohde J Müller S Wöste M Khanam T. Reconstructing Clonal Evolution in Relapsed and non-Relapsed Burkitt Lymphoma. Leukemia (2021) 35:639–43. doi: 10.1038/217s41375-020-0862-5218 PMC8318876 32404974

[17] R Core Team. R: A Language and Environment for Statistical Computing. Vienna, Austria: R Foundation for Statistical Computing (2020).

[18] Miller CA McMichael J Dang HX Maher CA Ding L Ley TJ. Visualizing Tumor Evolution With the Fishplot Package for R. BMC Genomics (2016) 17. doi: 10.1186/s12864-016-3195-z203 PMC5100182 27821060

[19] Therneau TM. A Package for Survival Analysis in R. (2020), R package version 3.2-7. Available at: (https://CRAN.R-project.org/package=survival)

[20] Kassambara A Kosinski M Biecek P. Survminer: Drawing Survival Curves Using ‘Ggplot2’. (2021), R package version 0.4.9201. Avaialble at: https://cran.r-project.org/web/packages/survminer/

[21] Gandhi UH Cornell RF Lakshman A Gahvari ZJ McGehee E Jagosky MH. Outcomes of Patients With Multiple Myeloma Refractory to CD38-Targeted Monoclonal Antibody Therapy. Leukemia (2019) 33:2266–75. doi: 10.1038/s41375-019-0435-7 PMC6820050 30858549

[22] Brioli A Melchor L Titley I Vijayaraghavan G Stephens C Zeisig A. The Impact of Long-Term Lenalidomide Exposure on the Cellular Composition of Bone Marrow. Leuk Lymphoma (2014) 55:2665–68. doi: 10.3109/10428194.2014.900765 24660850

[23] Dutta AK Alberge J-B Sklavenitis-Pistofidis R Lightbody ED Getz G Ghobrial IM. Single-Cell Profiling of Tumour Evolution in Multiple Myeloma - Opportunities for Precision Medicine. Nat Rev Clin Oncol (2022) 19:223–36. doi: 10.1038/s41571-021-00593-y 35017721

[24] Melchor L Brioli A Wardell CP Murison A Potter NE Kaiser MF. Single-Cell Genetic Analysis Reveals the Composition of Initiating Clones and Phylogenetic Patterns of Branching and Parallel Evolution in Myeloma. Leukemia (2014) 28:1705–15. doi: 10.1038/leu.2014.13 24480973

[25] Shah V Johnson DC Sherborne AL Ellis S Aldridge FM Howard-Reeves J. Subclonal TP53 Copy Number is Associated With Prognosis in Multiple Myeloma. Blood (2018) 132:2465–69. doi: 10.1182/blood-2018-06-857250 PMC6533595 30373884

[26] Shen YJ Mishima Y Shi J Sklavenitis-Pistofidis R Redd RA Moschetta M. Progression Signature Underlies Clonal Evolution and Dissemination of Multiple Myeloma. Blood (2021) 137:2360–72. doi: 10.1182/blood.2020005885 PMC8085483 33150374

[27] Walker BA Wardell CP Melchor L Brioli A Johnson DC Kaiser MF. Intraclonal Heterogeneity is a Critical Early Event in the Development of Myeloma and Precedes the Development of Clinical Symptoms. Leukemia (2014) 28:384–90. doi: 10.1038/leu.2013.199 PMC3916874 23817176

[28] Rasche L Chavan SS Stephens OW Patel PH Tytarenko R Ashby C. Spatial Genomic Heterogeneity in Multiple Myeloma Revealed by Multi-Region Sequencing. Nat Commun (2017) 8. doi: 10.1038/s41467-017-00296-y PMC5559527 28814763

[29] Merz M Merz AMA Wang J Wei L Hu Q Hutson N. Deciphering Spatial Genomic Heterogeneity at a Single Cell Resolution in Multiple Myeloma. Nat Commun (2022) 13. doi: 10.1038/s41467-022-28266-z PMC8831582 35145077

[30] Avet-Loiseau H Lannes R Perrot A Mazzotti C Divoux M Cazaubiel T. In Multiple Myeloma, High-Risk Secondary Genetic Events Observed at Relapse Are Present From the Diagnosis in Tiny Undetectable Subclones. Blood (2021) 138(Supplement 1):77. doi: 10.1182/blood-2021-146869

[31] Stengel A Kern W Haferlach T Meggendorfer M Fasan A Haferlach C. The Impact of TP53 Mutations and TP53 Deletions on Survival Varies Between AML, ALL, MDS and CLL: An Analysis of 3307 Cases. Leukemia (2017) 31:705–11. doi: 10.1038/leu.2016.263 27680515

[32] Carrasco DR Tonon G Huang Y Zhang Y Sinha R Feng B. High-Resolution Genomic Profiles Define Distinct Clinico-Pathogenetic Subgroups of Multiple Myeloma Patients. Cancer Cell (2006) 9:313–25. doi: 10.1016/j.ccr.2006.03.019 16616336

[33] Hanamura I Stewart JP Huang Y Zhan F Santra M Sawyer JR. Frequent Gain of Chromosome Band 1q21 in Plasma-Cell Dyscrasias Detected by Fluorescence *in Situ* Hybridization: Incidence Increases From MGUS to Relapsed Myeloma and is Related to Prognosis and Disease Progression Following Tandem Stem-Cell Transplantation. Blood (2006) 108:1724–32. doi: 10.1182/blood-2006-03-009910 PMC1895503 16705089

[34] Marchesini M Ogoti Y Fiorini E Samur AA Nezi L D’Anca M. ILF2 Is a Regulator of RNA Splicing and DNA Damage Response in 1q21-Amplified Multiple Myeloma. Cancer Cell (2017) 32:88–100. doi: 10.1016/j.ccell.2017.05.011 28669490 PMC5593798

[35] Landau DA Carter SL Getz G Wu CJ. Clonal Evolution in Hematological Malignancies and Therapeutic Implications. Leukemia (2014) 28:34–43. doi: 10.1038/leu.2013.248 23979521 PMC3934006

