# Supplementary material for: The Role of Clonal Evolution on Progression, Blood Parameters, and Response to Therapy in Multiple Myeloma
Source: Front Oncol. 2022 Jul 19;12:919278. doi: 10.3389/fonc.2022.919278 (PMC9343617; doi:10.3389/fonc.2022.919278)
Supplement: Supplementary file 1 [file DataSheet_1.pdf]

Supplementary Material

1 Supplementary Tables

Supplementary Table 1: Detailed overview of basic patient characteristics. Data for 25 patients with Multiple myeloma was available. Based on the presence of new clones emerging in the course of disease, we defined two subgroups: 1) without new clone (patients UPN05 to 16), 2) with new clone (patients UPN17 to 25). Due to presence of only 1 time point, patients UPN01 to 04 are excluded from subgroup analysis; SCT – stem cell transplantation.

Patient ID	Sex	Age at first diagnosis	Follow-up [years]	#FISH	SCT	Died
UPN01	F	60	1.33	1	no	no
UPN02	M	65	1.41	1	no	no
UPN03	F	64	1.75	1	no	no
UPN04	M	67	12.5	1	no	no
UPN05	M	73	1.41	2	no	no
UPN06	M	67	2.41	2	no	no
UPN07	M	53	3.33	3	no	no
UPN08	F	65	3.41	7	yes	no
UPN09	F	38	3.62	4	yes	yes
UPN10	M	48	3.92	2	no	no
UPN11	M	53	4.25	6	yes	no
UPN12	M	45	4.50	2	no	no
UPN13	F	53	4.67	2	no	no
UPN14	F	57	5.00	7	yes	no
UPN15	F	70	5.22	2	no	yes
UPN16	M	50	17.76	2	no	no
UPN17	F	56	1.76	5	yes	yes
UPN18	F	58	2.00	3	yes	no
UPN19	M	48	2.01	3	no	yes
UPN20	M	59	2.25	3	no	no
UPN21	M	60	3.41	3	no	no
UPN22	M	58	5.00	11	yes	no
UPN23	F	59	5.01	2	no	yes
UPN24	M	53	5.50	11	yes	no
UPN25	M	49	6.42	6	yes	no

Supplementary Table 2: Summarized main characteristics of the study population and the two subgroups analyzed; IQR – interquartile range; SCT – stem cell transplantation.

	Study population UPN01 to 25	Subgroup 1 UPN05 to 16	Subgroup 2 UPN17 to 25
n	25	12	9
Male:Female	15:10	7:5	6:3
Age at first diagnosis (median [IQR])	58 [53-64]	53 [49.5-65.5]	58 [53-59]
Follow-up [years] (median [IQR])	3.62 [2.01-5]	4.09 [3.39-4.75]	3.41 [2.01-5.01]
#FISH (median [range])	3 [1-11]	2 [2-7]	3 [3-11]
#Patients with SCT	9	4	5
#Patients died	5	2	3

Supplementary Table 3: Overview of cytogenetic aberrations, their CCFs and associated clones for every patient and time point. Presence and absence of cytogenetic aberrations was analyzed by FISH. Absence is indicated by a negative result (neg.). CCF is based on the number of cells with aberration divided by the total number of cells analyzed. In case of no analysis being performed, the corresponding cell in the table is left empty. Every aberration is associated with a clone. A parent defines the precursor clone. A missing precursor, i.e. development from normal cells, is indicated by '-'.

UPN01

Aberration	TP1	Clone	Parent
del(17)(p13)	98%	1	-
del(4)(p16)	96%	2	1
del(16)(q23)	96%	2	1
dup(1)(q21)	96%	2	1
del(1)(p32.3)	96%	2	1
+3	93%	3	2
dup(1)(q21)x2	87%	4	3

UPN02

Aberration	TP1	Clone	Parent
del(13)(q14)	99%	1	-
dup(15)(q22)	99%	1	-
dup(1)(q21)x2	99%	1	-
dup(17)(p13)	92%	2	1
rear(14)(q32.3)	90%	2	1
dup(19)(q13)	77%	3	2

UPN03

Aberration	TP1	Clone	Parent
t(11;14)	83%	1	-
rear(14)(q32)	69%	2	1
+15	13%	3	2

UPN04

Aberration	TP1	Clone	Parent
-	-	-	-

UPN05

Aberration	TP1	TP2	Clone	Parent
del(20)(q12)	81%	neg.	1	-
dup(11)(q22.3)	90%	neg.	2	1
dup(11)(q13)	80%	neg.	2	1
dup(11)(q22.3)x4	15%	neg.	3	2
dup(11)(q13)x4	13%	neg.	3	2
dup(17p)x2	15%	neg.	3	2
dup(16)(q23)x2	11%	neg.	3	2
dup(14)(q32)x2	11%	neg.	3	2
dup(4)(p16)x2	10%	neg.	3	2

UPN06

Aberration	TP1	TP2	Clone	Parent
dup(1)(q21)	98%	neg.	1	-
t(11;14)	99%	neg.	1	-
del(13q)	98%	neg.	1	-
rear(14)(q32.33)	98%	neg.	1	-
del(16)(q22)	97%	neg.	1	-

UPN07

Aberration	TP1	TP2	TP3	Clone	Parent
dup(1)(q21)	97%	neg.	95%	1	-
t(14;20)	98%	neg.	96%	1	-
dup(7)(p11.1q11.1)	89%	neg.	95%	2	1
rear(14)(q32.33)	98%	neg.	neg.	3	2

UPN08

Aberration	TP1	TP2	TP3	TP4	TP5	TP6	TP7	Clone	Parent
dup(1)(q21)	85%	neg.	neg.	neg.	neg.	neg.	neg.	1	-
t(4;14)	84%	neg.	neg.	neg.	neg.	neg.	neg.	1	-
del(13q)	80%	neg.	neg.	neg.	neg.	neg.	neg.	2	1

UPN09

Aberration	TP1	TP2	TP3	TP4	Clone	Parent
del(1)(p32)	80%	neg.	80%	67%	1	-
del(1)(q21)	80%	neg.	80%	67%	1	-
del(14)(q32)	80%	neg.	78%	68%	1	-
-17	79%	neg.	85%	56%	2	1
del(13)(q14)	74%	neg.	78%	56%	3	2

del(13)(q34)	74%	neg.	78%	56%	3	2

UPN10

Aberration	TP1	TP2	Clone	Parent
dup(3)(p11.1q11.1)	7%	15%	1	-
dup(9)(q12)	14%	13%	2	-
del(15)(p11.1q11.1)	15%	neg.	3	-

UPN11

Aberration	TP1	TP2	TP3	TP4	TP5	TP6	Clone	Parent	
								V1	V2
del(13q)	95%	6%	3%	neg.	9%	74%	1	-	-
del(17)(p13.1)	96%	neg.	4%	neg.	8%	73%	1	-	-
dup(9)(q12)	94%	8%	neg.	neg.	3%	80%	2	1	1
dup(15)(p11.1q11.1)	94%	8%	neg.	neg.	3%	71%	2	1	1
dup(7)(p11.1q11.1)	89%	8%	neg.	neg.	7%	68%	3	2	2
dup(15)(p11.1q11.1)x2	20%	neg.	neg.	neg.	neg.	33%	4	3	3
dup(1)(q21)	9%	neg.	neg.	neg.			5	4	3

UPN12

Aberration	TP1	TP2	Clone	Parent
dup(11)(q22)	70%	15%	1	-
t(11;14)	68%	10%	2	1
rear(14)(q32)	64%	neg.	3	2

UPN13

Aberration	TP1	TP2	Clone	Parent			
				V1	V2	V3	V4
dup(1)(q21)	45%	neg.	1	-	-	-	-
rear(14)(q32.33)	40%	neg.	2	1	1	-	-
del(13)(q14q32)	32%	neg.	3	2	-	2	1

UPN14

Aberration	TP1	TP2	TP3	TP4	TP5	TP6	TP7	Clone	Parent
dup(9)(q12)	20%	neg.	neg.					1	-
dup(15)(p11.1q11.1)	20%	neg.	neg.					1	-
del(17)(p13.1)	18%	74%	62%	neg.	neg.	neg.	neg.	2	-
dup(17)(q11.2)	19%	80%	77%					3	2
dup(17)(q22)	17%	80%	65%					3	2
dup(17)(q22)x3	10%	80%	35%					4	3
dup(3)(p11.1q11.1)	12%	61%						4	3

UPN15

Aberration	TP1	TP2	Clone	Parent
del(13q)	33.3%	neg.	1	-
+17, +17	23.8%	16%	2	-

UPN16

Aberration	TP1	TP2	Clone	Parent
dup(11)(q13.3)x2	39%	98%	1	-
dup(14)(q32.33)x1-2		94%	2	1
dup(1)(q21)		16%	3	2

UPN17

Aberration	TP1	TP2	TP3	TP4	TP5	Clone	Parent
t(4;14)(p16.3q32.33)	96%	90%	80%	neg.	neg.	1	-
-13	neg.	neg.	80%	3%	neg.	2	1
dup(1)(q21)	2%	neg.	54%	14%	neg.	3	-

UPN18

Aberration	TP1	TP2	TP3	Clone	Parent
del(13)(q14)	98%	95%	neg.	1	-
dup(9)(q34)	84%	96%	neg.	2	1
dup(15)(q22)	84%	97%	neg.	2	1
dup(5)(p15q35)		94%	neg.	3	2
dup(19)(q13)	77%	89%	neg.	4	3
dup(4)(p16)	73%	89%	neg.	5	4
rear(8)(q24)	16%	21%	neg.	6	5
dup(14)(q32)	8%	1%	neg.	7	6
dup(1)(q21.3)	4%	neg.	neg.	8	7
del(16)(q23)	neg.	10%	neg.	9	6
del(17)(p13)	neg.	4%	neg.	10	9

UPN19

Aberration	TP1	TP2	TP3	Clone	Parent
t(4;14)	98%	100%	28%	1	-
del(13q)	100%	90%	28%	1	-
rear(14)(q32.33)	96%		neg.	2	1
+3	79%		neg.	3	2
-15	21%	14%		4	3

+9	neg.	15%		5	4
del(17p)	neg.	neg.	28%	6	1
del(1)(p32)	neg.	neg.	18%	9	8
dup(1)(q21)	neg.	neg.	15%	7	6
dup(1)(q21)x2	neg.	neg.	3%	8	7

UPN20

Aberration	TP1	TP2	TP3	Clone	Parent
del(13)(q14)	30%	93%	neg.	1	-
t(4;14)	20%	89%	neg.	2	1
rear(14)(q32)		90%	neg.	2	1
del(16)(q23)	23%	70%	neg.	3	2
dup(9)(q34)	neg.	72%	neg.	4	3
del(17)(p13)	neg.	10%	neg.	5	4

UPN21

Aberration	TP1	TP2	TP3	Clone	Parent
rear(14)(q32)	89%	neg.	neg.	1	-
dup(1)(q21)	81%	neg.		2	1
del(13q)	15%	neg.	93%	3	-
dup(16)(q23)	neg.	neg.	96%	4	3
t(11;14)		neg.	84%	5	4
dup(1)(q32)	neg.	neg.	82%	5	4

UPN22

Aberration	TP1	TP2	TP3	TP4	TP5	TP6	TP7	TP8	TP9	TP10	TP11	Clone	Parent
t(11;14)	99%	4%	neg.	neg.	neg.	neg.	neg.	neg.	neg.			1	-
del(17p)	80%	neg.	neg.	neg.	neg.	neg.	neg.	neg.	neg.	neg.	neg.	2	1
-15		4%	neg.	neg.	neg.	8%	5%	6%	neg.	3%	11%	3	-

UPN23

Aberration	TP1	TP2	Clone	Parent
t(11;14)	neg.	91%	1	-
dup(1)(q21)	neg.	80%	2	1
dup(1)(q21)x2	neg.	66%	3	2

UPN24

Aberration	TP1	TP2	TP3	TP4	TP5	TP6	TP7	TP8	TP9	TP10	TP11	Clone	Parent
t(11;14)	13%	6%	26%	neg.	neg.	neg.	neg.	neg.	neg.	neg.	neg.	1	-
dup(11)(q13)	13%	0%	26%	neg.	neg.	neg.	neg.	neg.	neg.	neg.	neg.	2	-
rear(14)(q32)	8%	8%		neg.	neg.	neg.	neg.	neg.	neg.	neg.	neg.	3	-
del(13)(q14)	neg.	10%	36%	neg.	neg.	neg.	neg.	neg.	neg.	neg.	neg.	4	-
dup(13q)	neg.	10%	36%	neg.	neg.	neg.	neg.	neg.	neg.	neg.	neg.	4	-
del(17)(p13)	neg.	4%	neg.	neg.	neg.	neg.	neg.	neg.	neg.	neg.	neg.	5	-

UPN25

Aberration	TP1	TP2	TP3	TP4	TP5	TP6	Clone	Parent	
								V1	V2
del(14)(q32.33)	86%	23%	neg.	83%	96%		1	-	-
del(13q)	91%		neg.	77%	93%		2	1	1
dup(1)(q21)	34%	12%	neg.	65%	90%		3	2	2
dup(1)(q21)x2	neg.	3%	neg.	23%	30%		4	3	3
dup(11)(q13q22.3)	neg.	2%				22%	5	4	-

2 Supplementary Figures

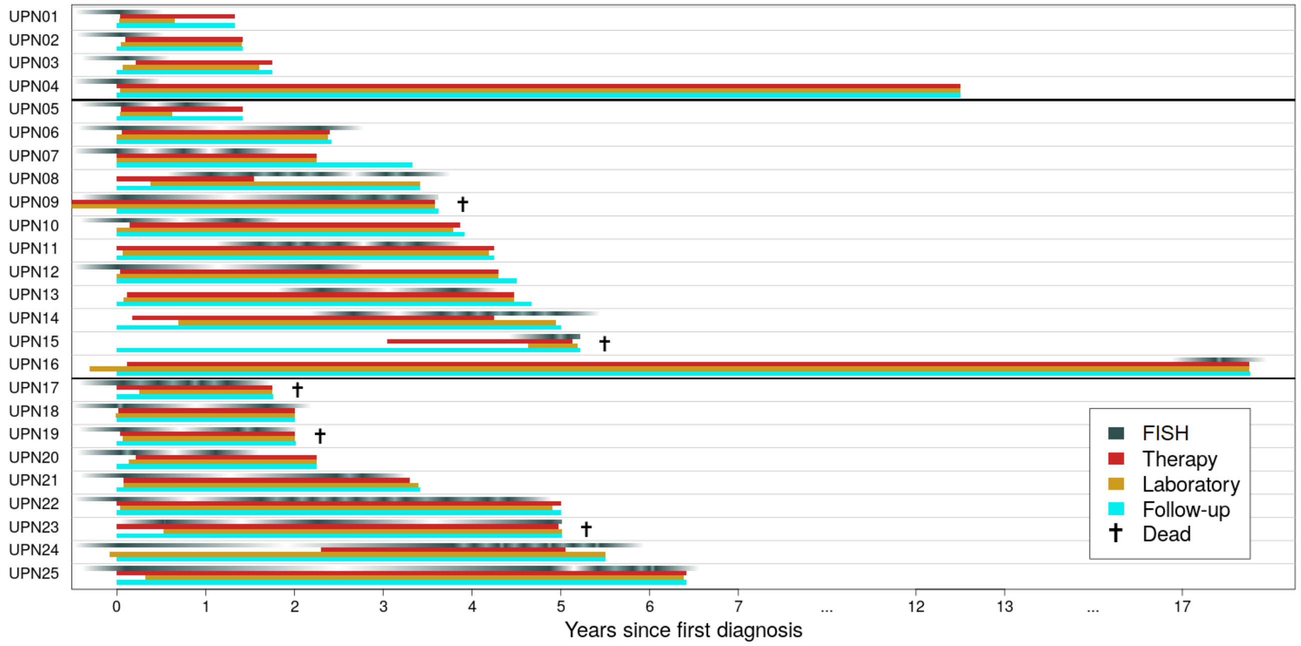

Supplementary Figure 1: Data available for every patient, including information on cytogenetic aberrations detected by FISH (green), therapy (red), laboratory parameters (yellow) and follow-up (light blue). For 4 out of 25 patients (UPN01 to 04), information on cytogenetic aberrations was available at only 1 time point. Remaining 21 patients were characterized by 2 to 11 FISH analyses. The group was split into patients without a new clone emerging in the course of disease (patients UPN05 to 16) and patients with a new clone (patients UPN17 to 25).

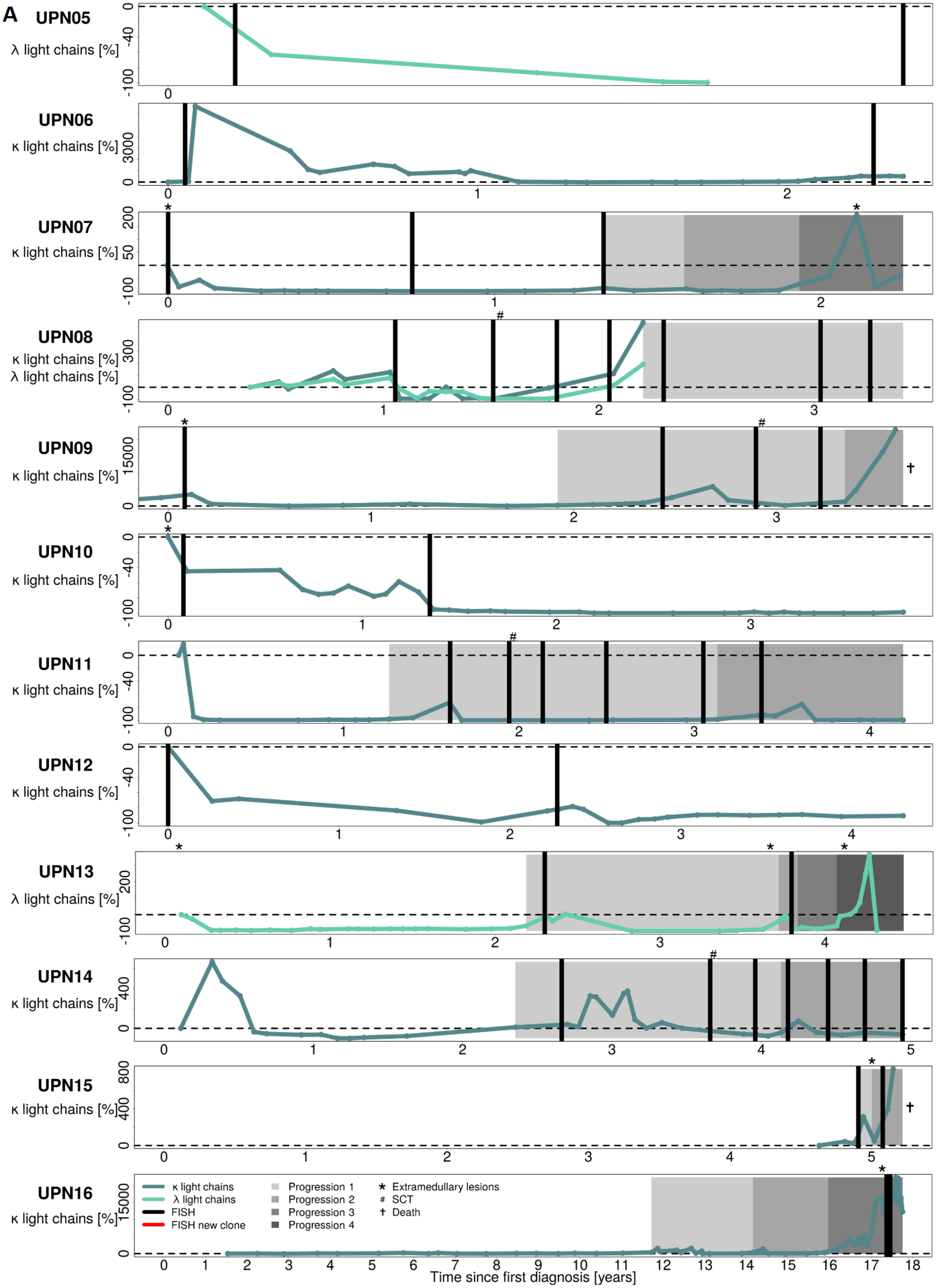
A
UPN05
λ light chains [%]
UPN06
κ light chains [%]
UPN07
κ light chains [%]
UPN08
κ light chains [%]
λ light chains [%]
UPN09
κ light chains [%]
UPN10
κ light chains [%]
UPN11
κ light chains [%]
UPN12
κ light chains [%]
UPN13
λ light chains [%]
UPN14
κ light chains [%]
UPN15
κ light chains [%]
UPN16
κ light chains [%]
κ light chains
λ light chains
FISH
FISH new clone
Progression 1
Progression 2
Progression 3
Progression 4
* Extramedullary lesions
# SCT
† Death
Time since first diagnosis [years]

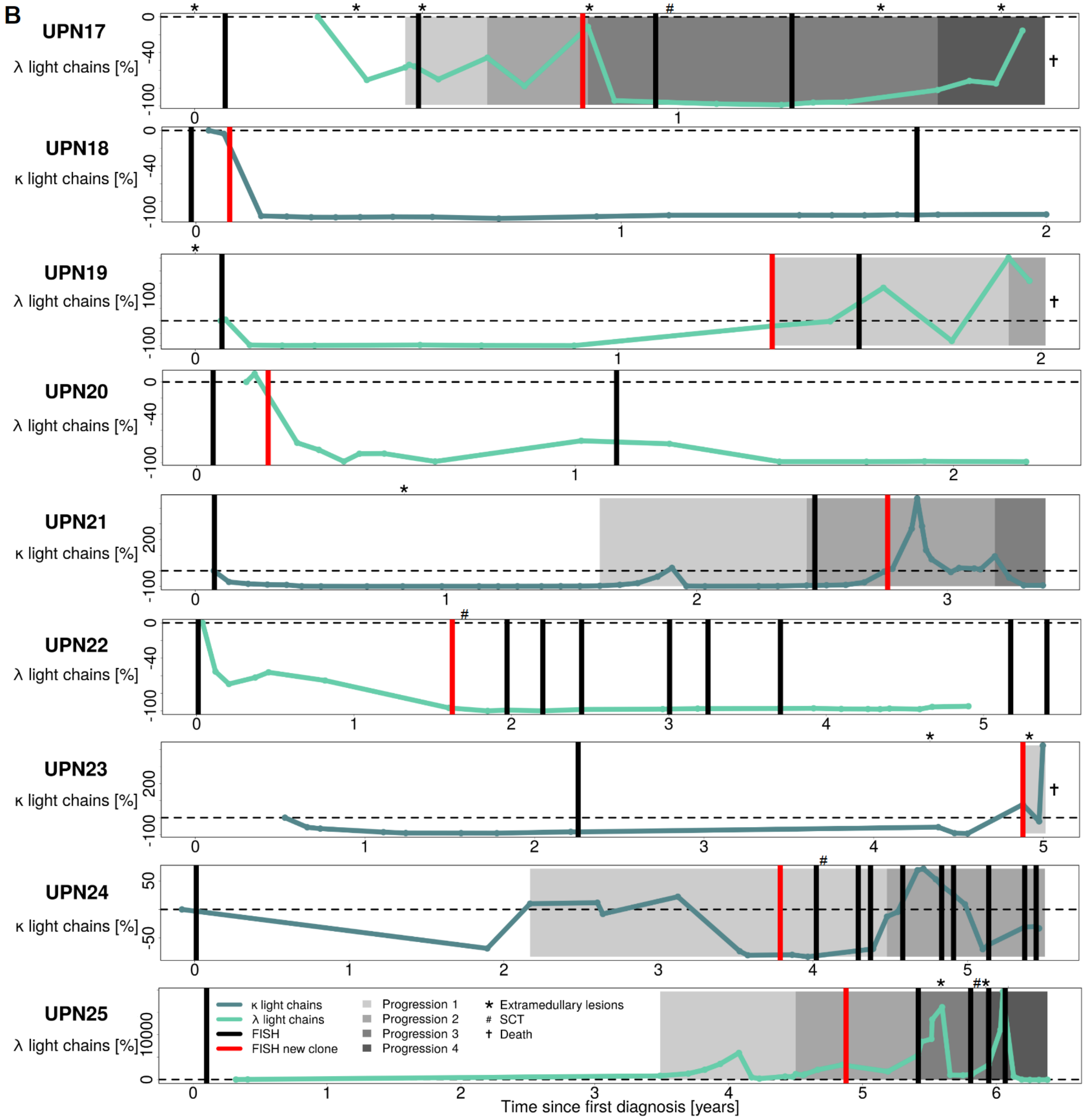

Supplementary Figure 2: Relative development of κ (dark green) and λ (light green) light chains in serum from first diagnosis until the end of follow-up. Horizontal dashed lines indicate the level of light chains at first measurement. A) Patients without a new clone emerging in the course of disease (patients UPN05 to 16). B) Patients with a new clone emerging in the course of disease (patients UPN17 to 25).

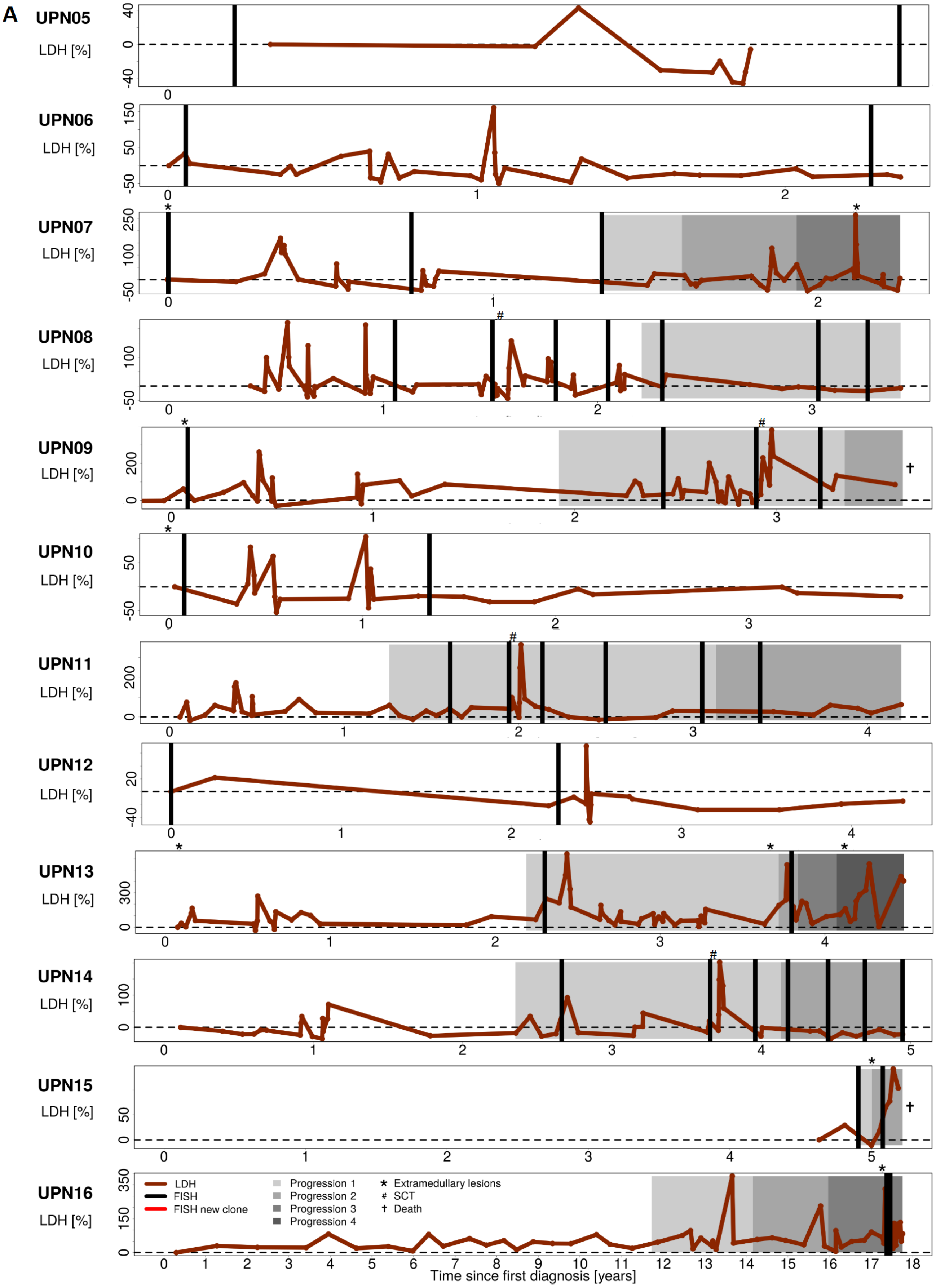
A
UPN05
LDH [%]
UPN06
LDH [%]
UPN07
LDH [%]
UPN08
LDH [%]
UPN09
LDH [%]
UPN10
LDH [%]
UPN11
LDH [%]
UPN12
LDH [%]
UPN13
LDH [%]
UPN14
LDH [%]
UPN15
LDH [%]
UPN16
LDH [%]
LDH
FISH
FISH new clone
Progression 1
Progression 2
Progression 3
Progression 4
* Extramedullary lesions
# SCT
† Death
Time since first diagnosis [years]

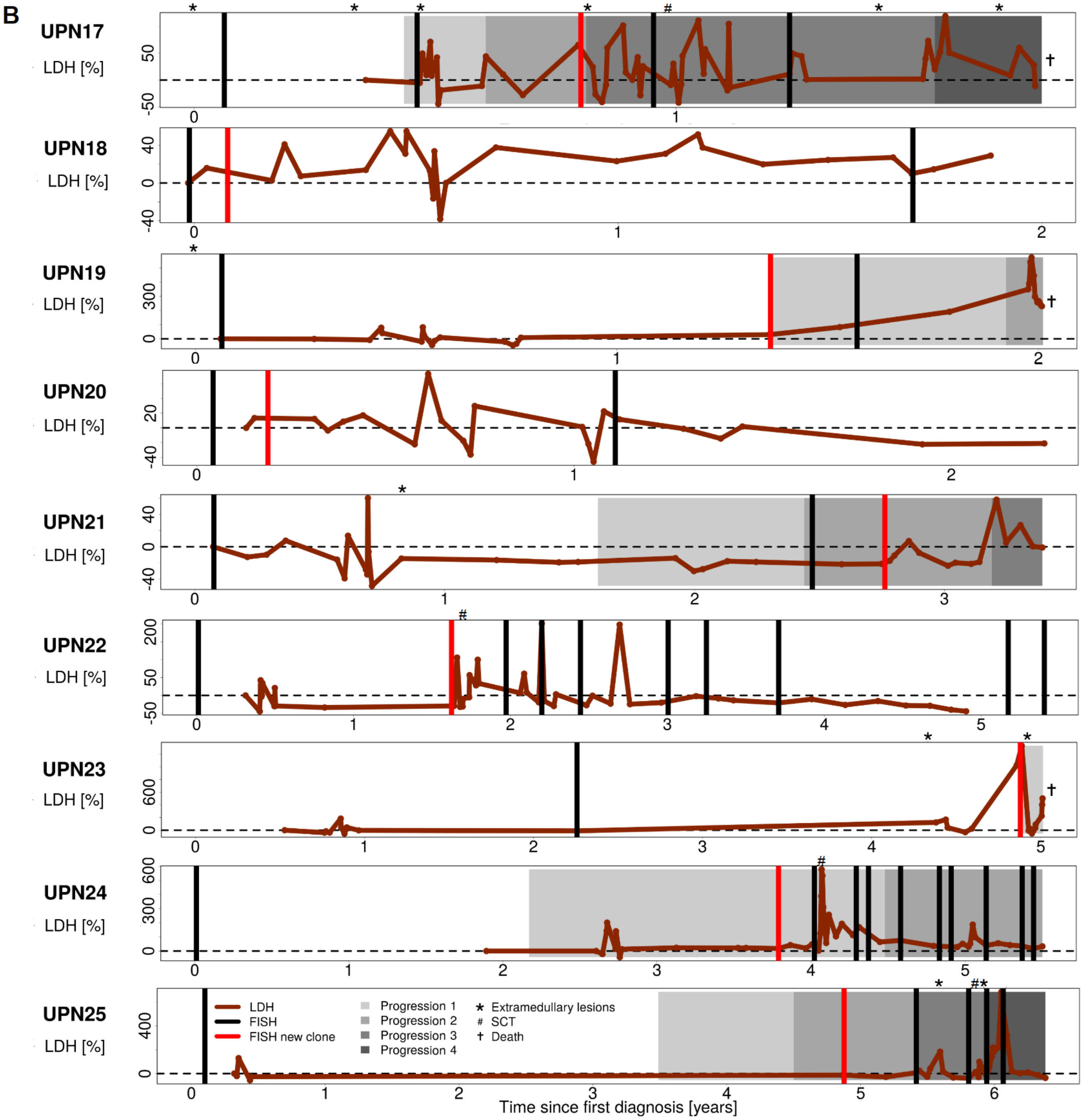

Supplementary Figure 3: Relative development of LDH activation from first diagnosis until the end of follow-up. Horizontal dashed lines indicate the level of LDH activation at first measurement. A) Patients without a new clone emerging in the course of disease (patients UPN05 to 16). B) Patients with a new clone emerging in the course of disease (patients UPN17 to 25).

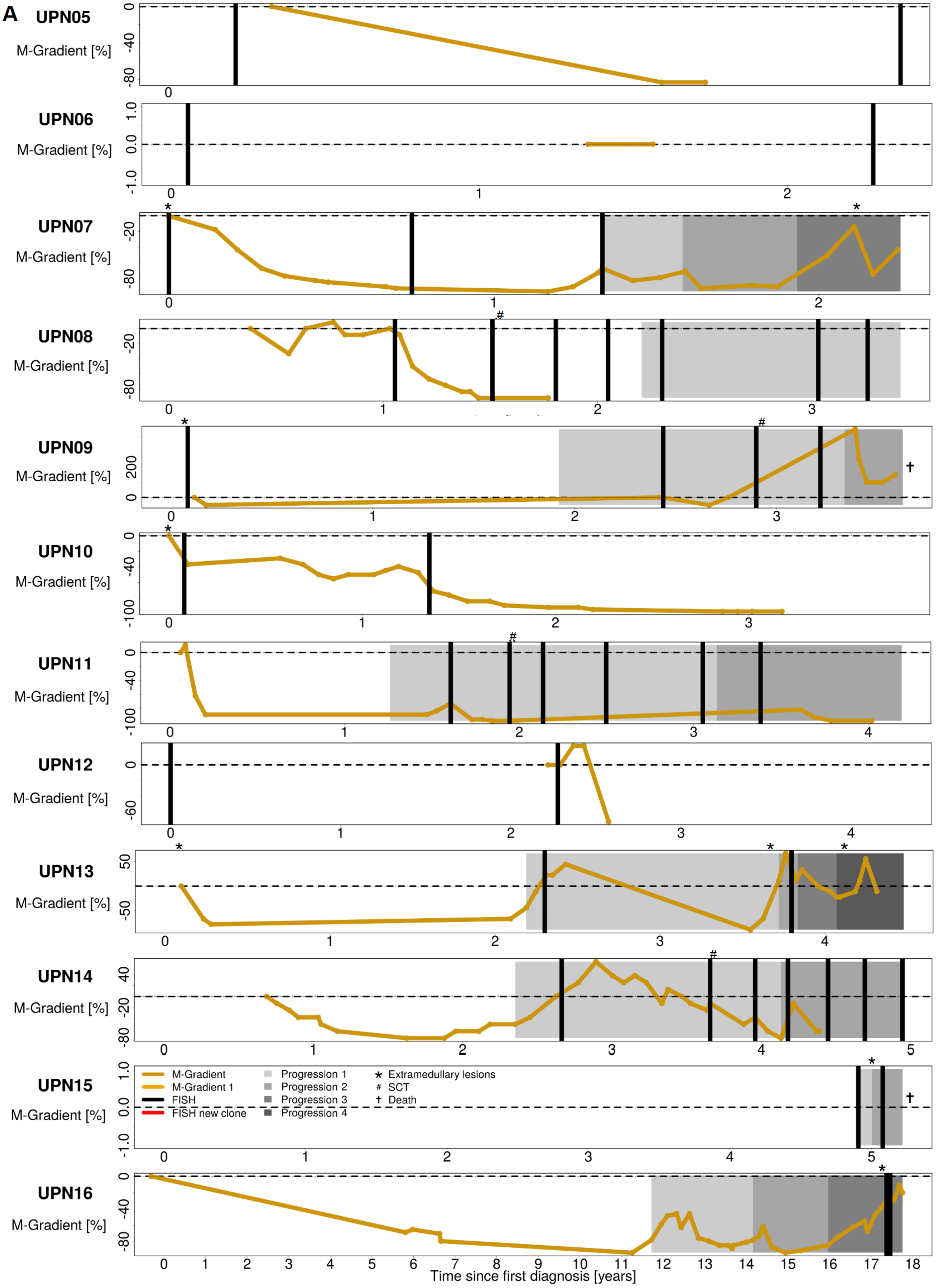
A
UPN05
UPN06
UPN07
UPN08
UPN09
UPN10
UPN11
UPN12
UPN13
UPN14
UPN15
UPN16
M-Gradient [%]
M-Gradient
M-Gradient 1
FISH
FISH new clone
Progression 1
Progression 2
Progression 3
Progression 4
* Extramedullary lesions
# SCT
† Death
Time since first diagnosis [years]

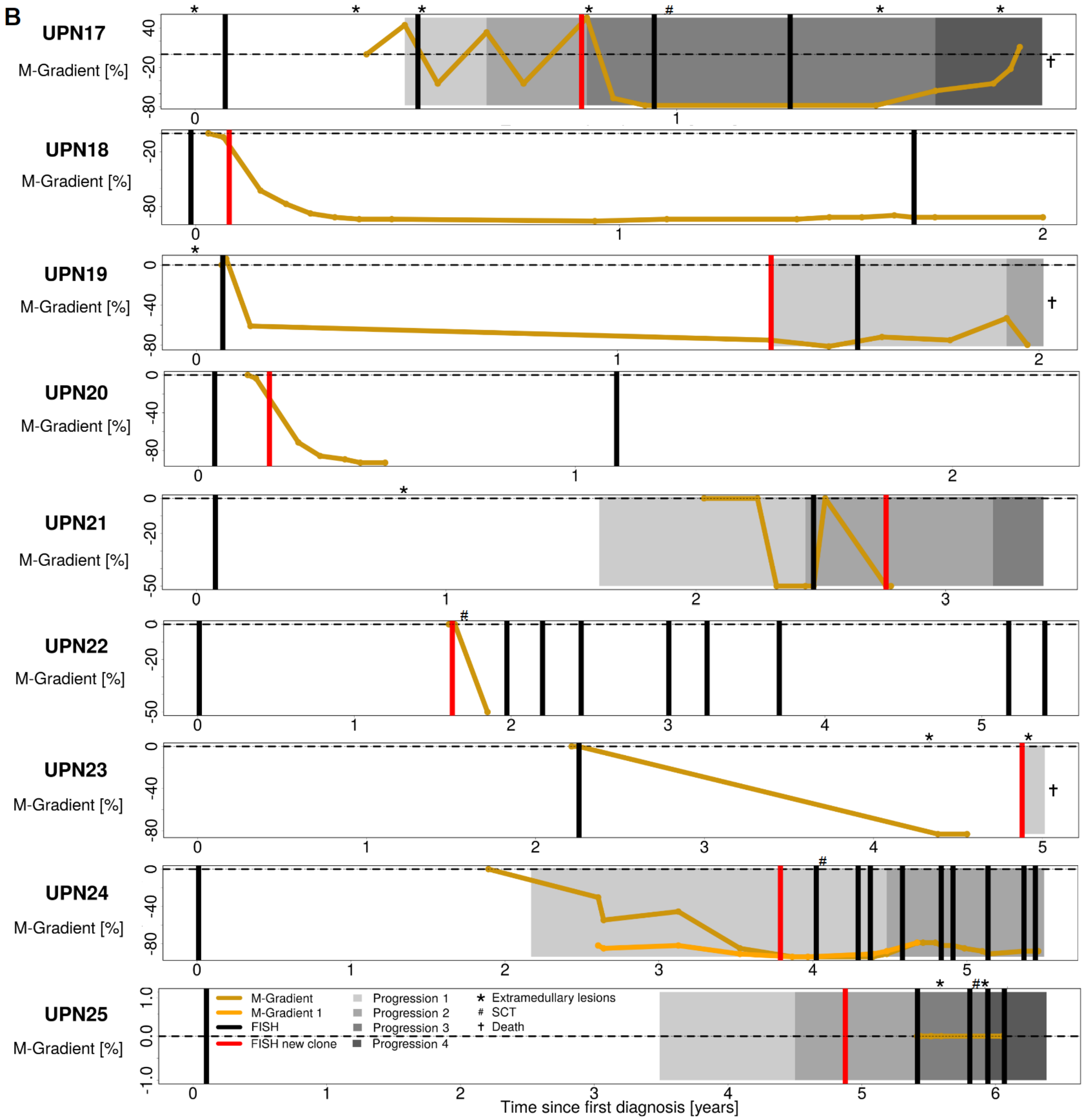

Supplementary Figure 4: Relative development of monoclonal protein (M-Gradient) from first diagnosis until the end of follow-up. Horizontal dashed lines indicate the level of LDH activation at first measurement. A) Patients without a new clone emerging in the course of disease (patients UPN05 to 16). B) Patients with a new clone emerging in the course of disease (patients UPN17 to 25).

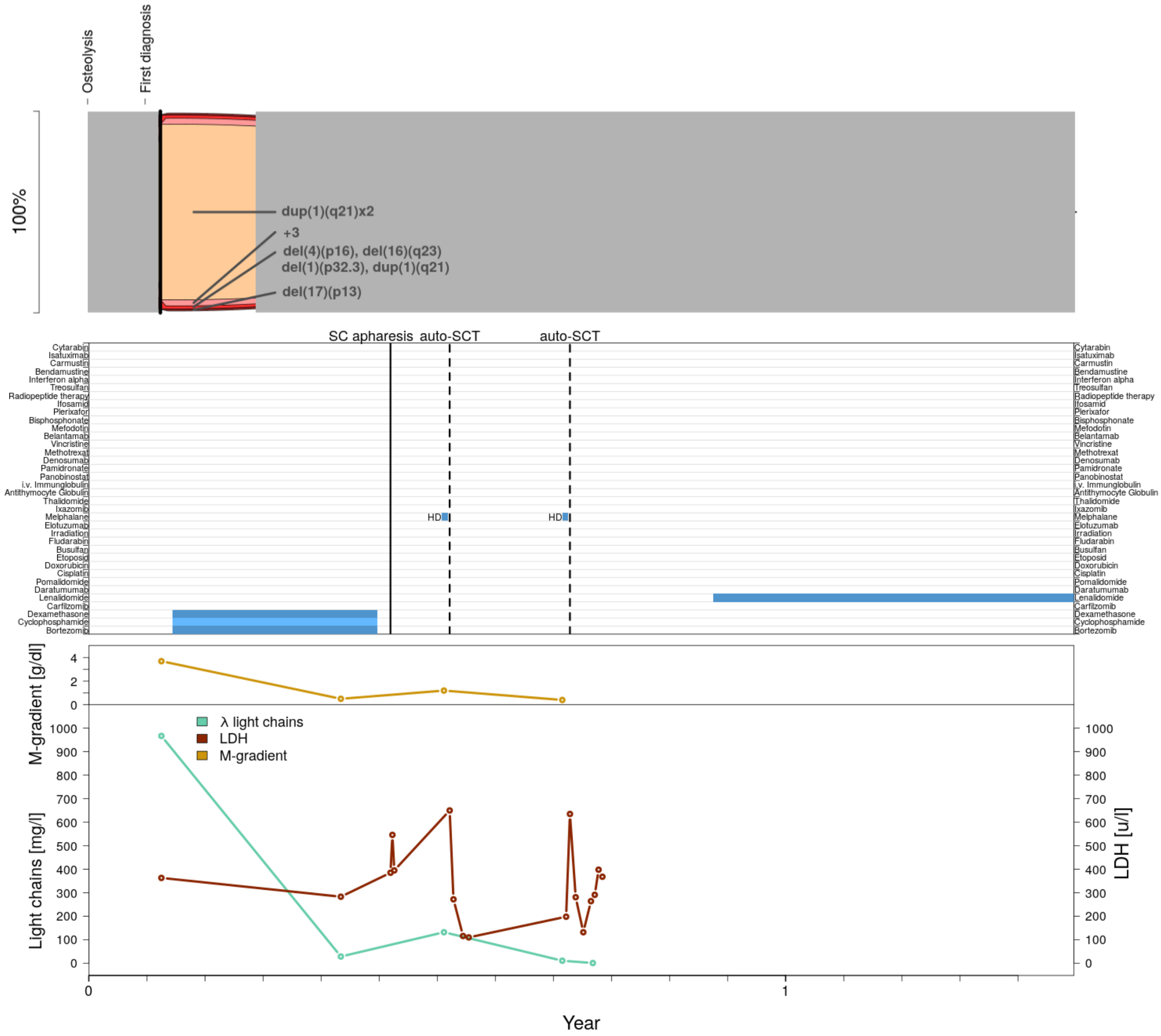

Supplementary Figure 5: Clonal evolution, applied therapies and development of laboratory parameters of patient UPN01. Vertical black line in the clonal evolution plot indicates the time point of aberration analysis; auto-SCT – autologous stem cell transplantation, HD – high dose, SC – stem cell.

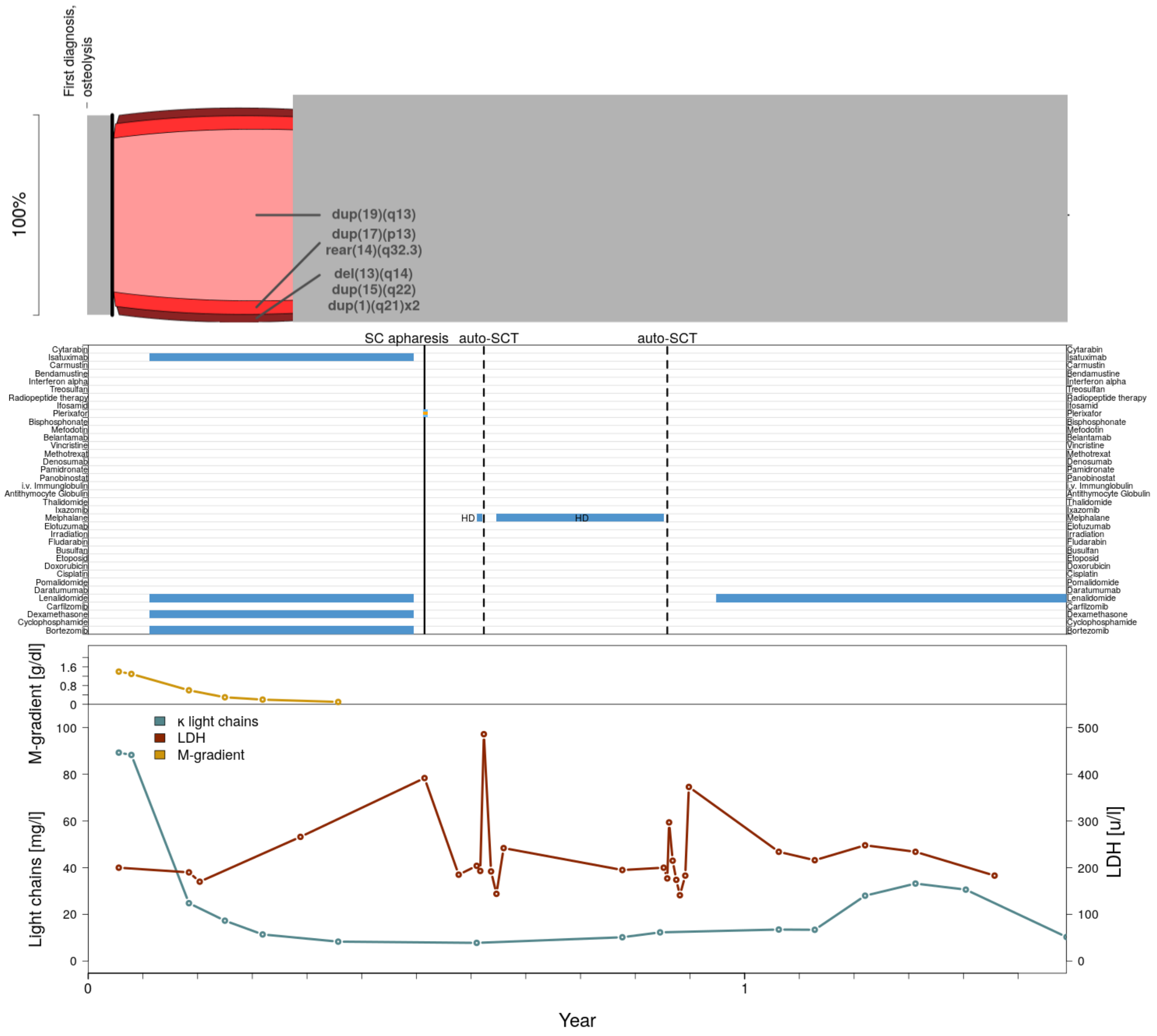

Supplementary Figure 6: Clonal evolution, applied therapies and development of laboratory parameters of patient UPN02. Vertical black line in the clonal evolution plot indicates the time point of aberration analysis; auto-SCT – autologous stem cell transplantation, HD – high dose, SC – stem cell.

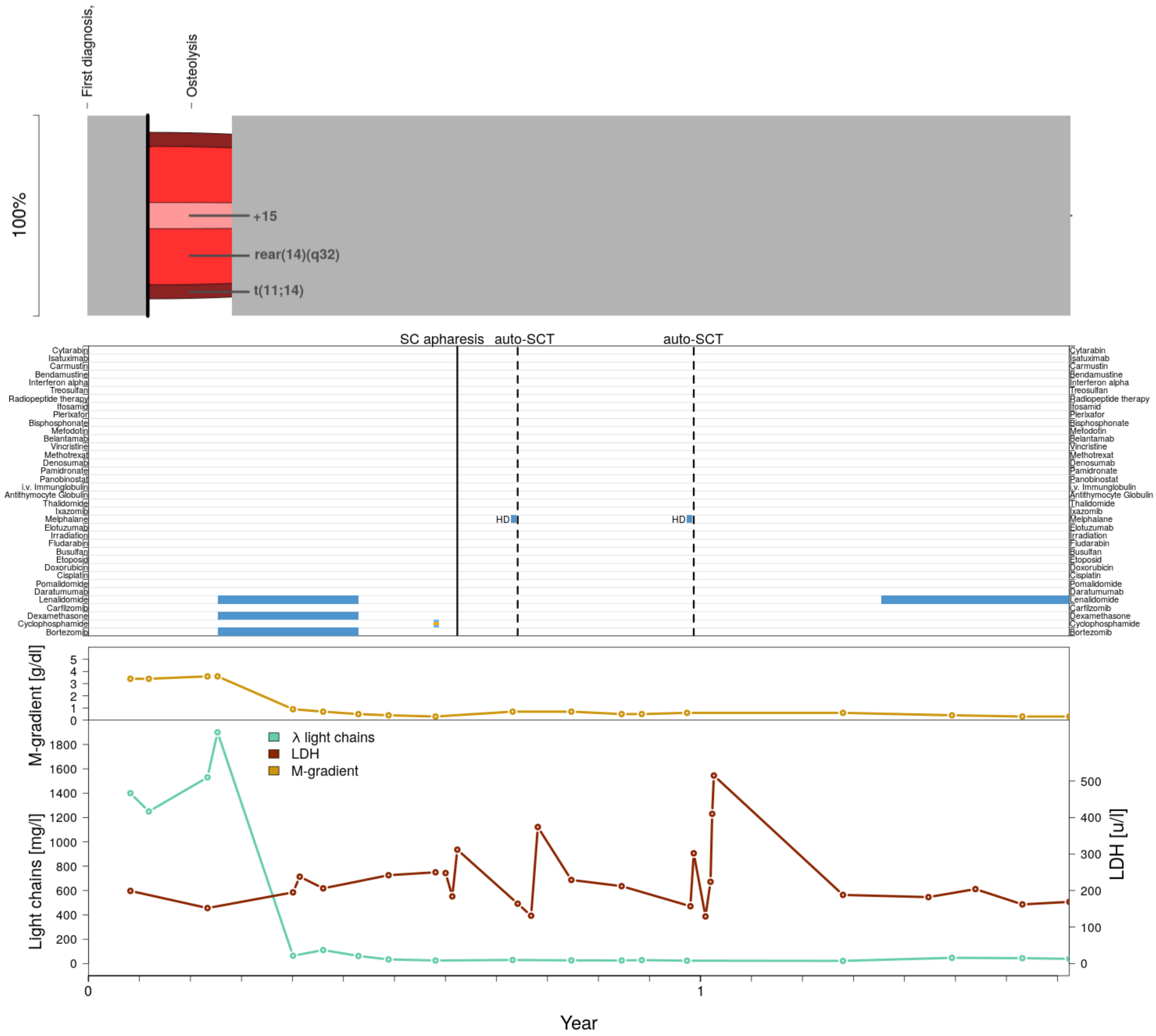

Supplementary Figure 7: Clonal evolution, applied therapies and development of laboratory parameters of patient UPN03. Vertical black line in the clonal evolution plot indicates the time point of aberration analysis; auto-SCT – autologous stem cell transplantation, HD – high dose, SC – stem cell.

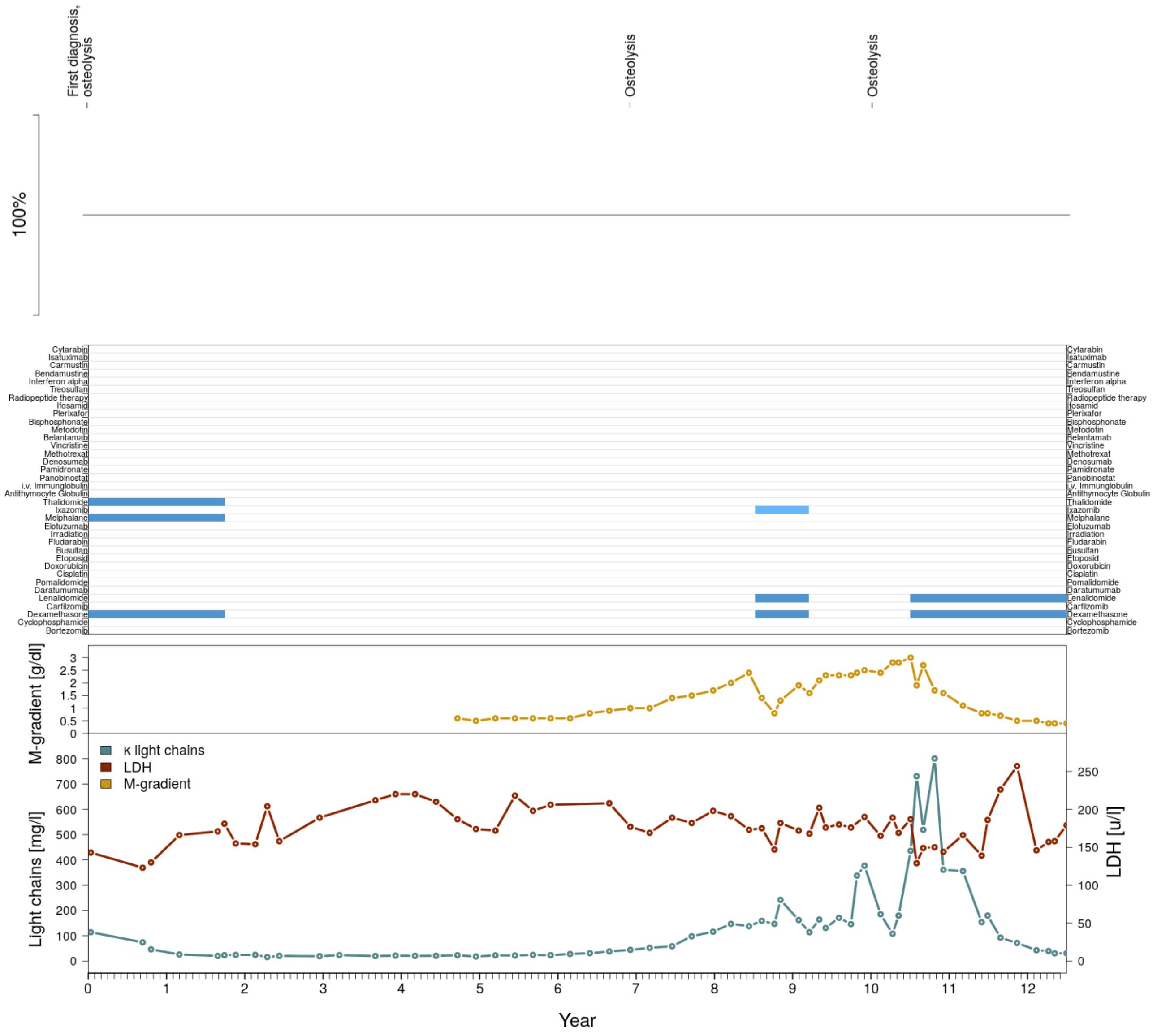

Supplementary Figure 8: Applied therapies and development of laboratory parameters of patient UPN04. No aberrations have been detected.

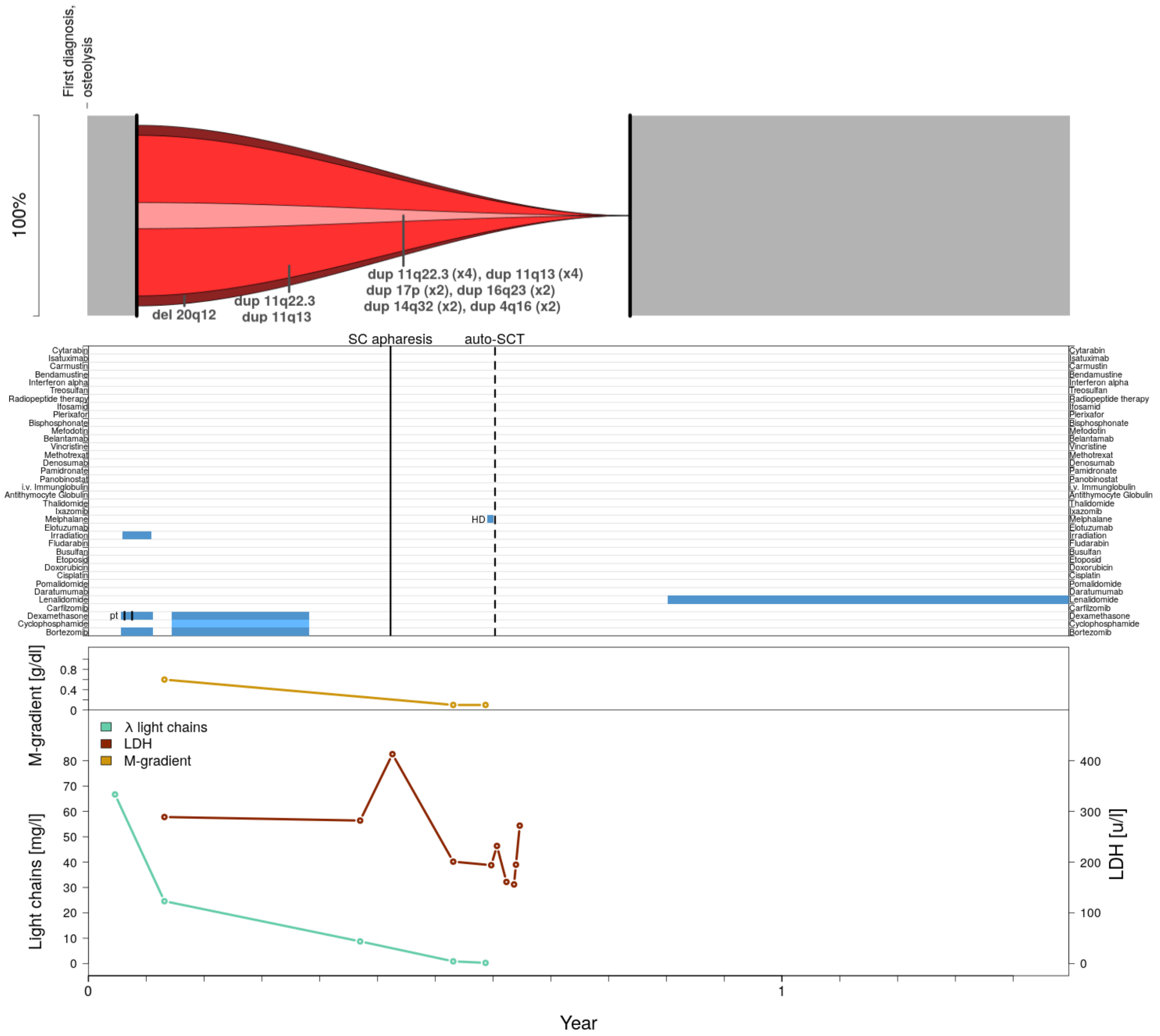

Supplementary Figure 9: Clonal evolution, applied therapies and development of laboratory parameters of patient UPN05. Vertical black lines in the clonal evolution plot indicate the time points of aberration analysis; auto-SCT – autologous stem cell transplantation, HD – high dose, pt – pulse therapy, SC – stem cell.

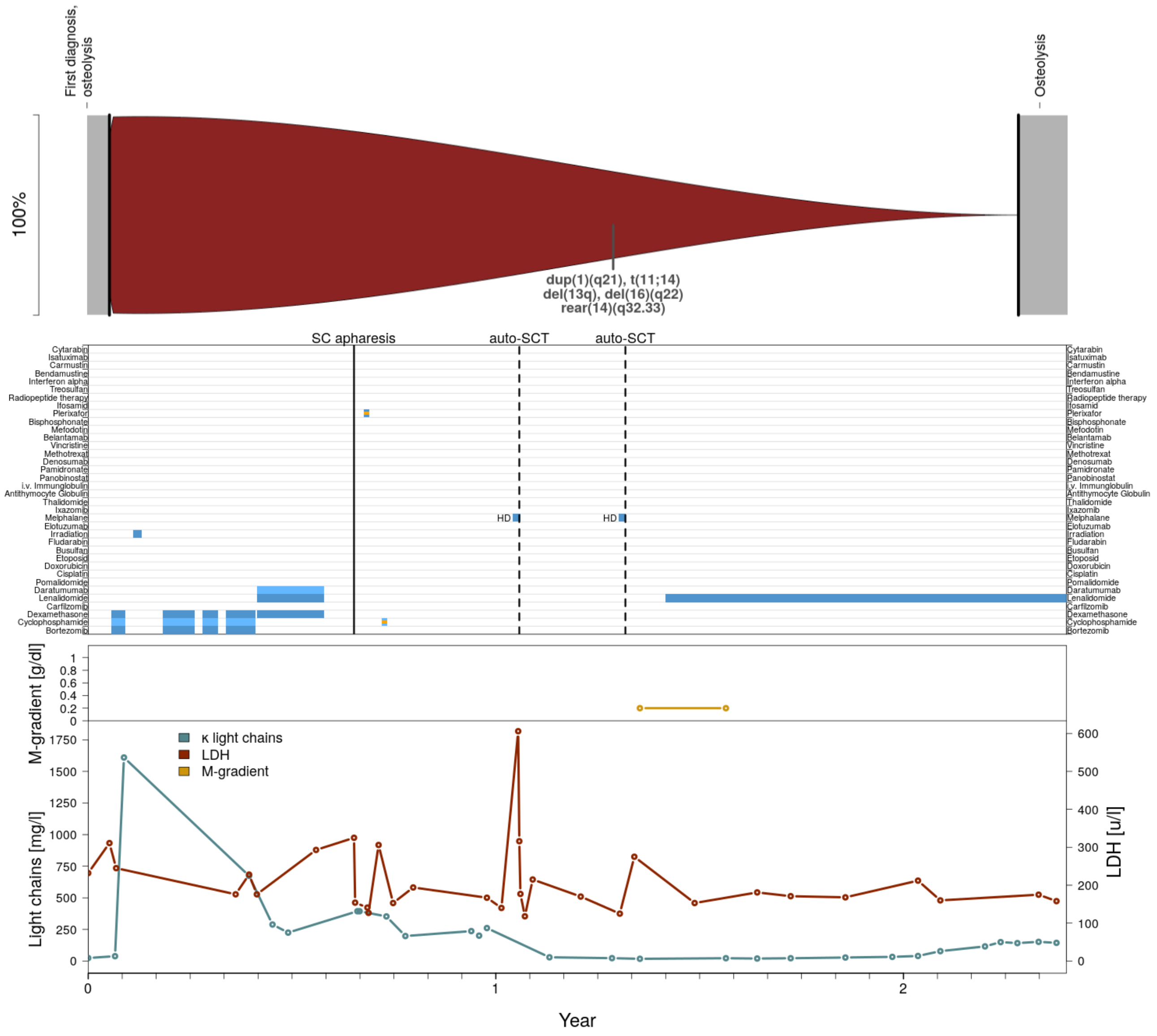

Supplementary Figure 10: Clonal evolution, applied therapies and development of laboratory parameters of patient UPN06. Vertical black lines in the clonal evolution plot indicate the time points of aberration analysis; auto-SCT – autologous stem cell transplantation, HD – high dose, SC – stem cell.

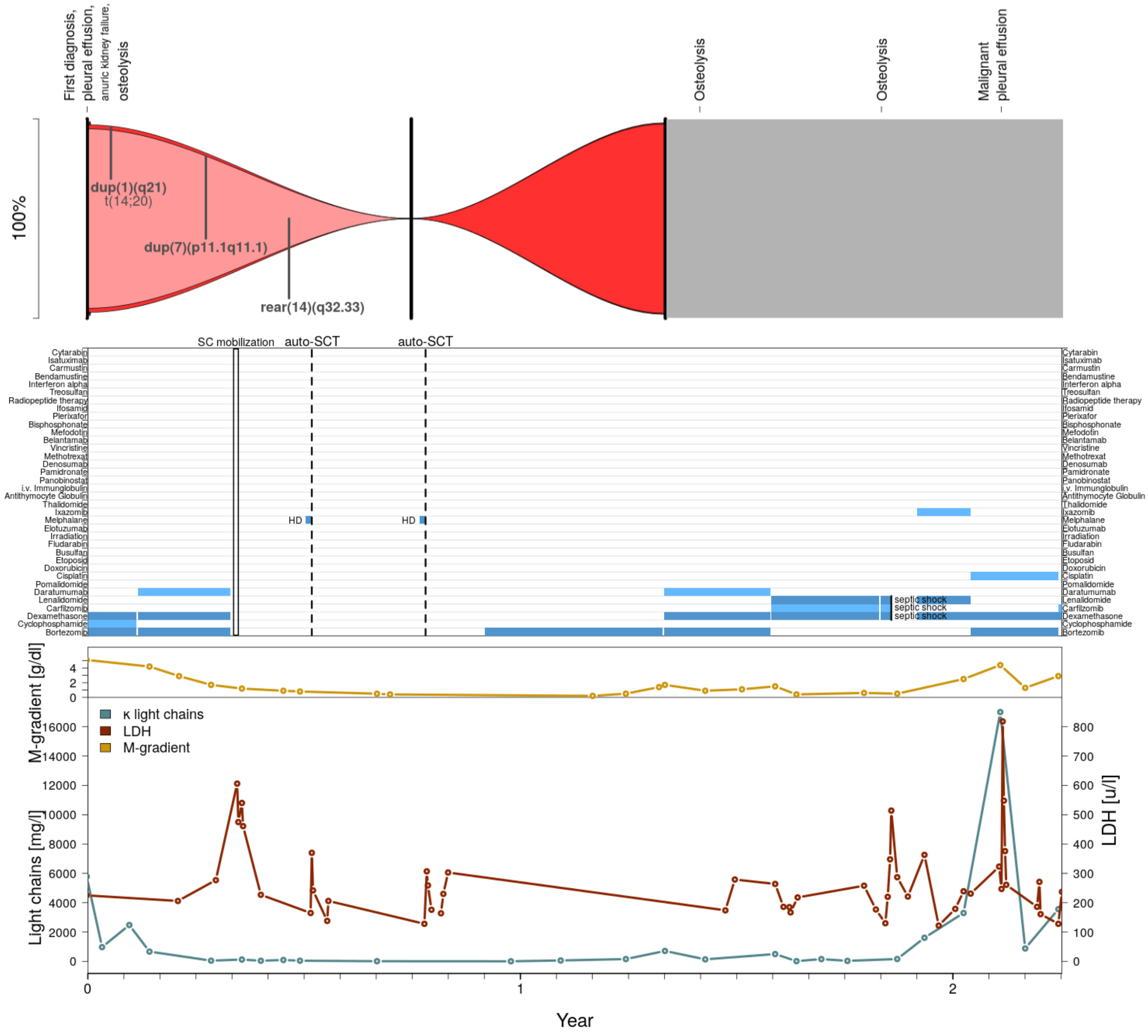

Supplementary Figure 11: Clonal evolution, applied therapies and development of laboratory parameters of patient UPN07. Vertical black lines in the clonal evolution plot indicate the time points of aberration analysis; auto-SCT – autologous stem cell transplantation, HD – high dose, SC – stem cell.

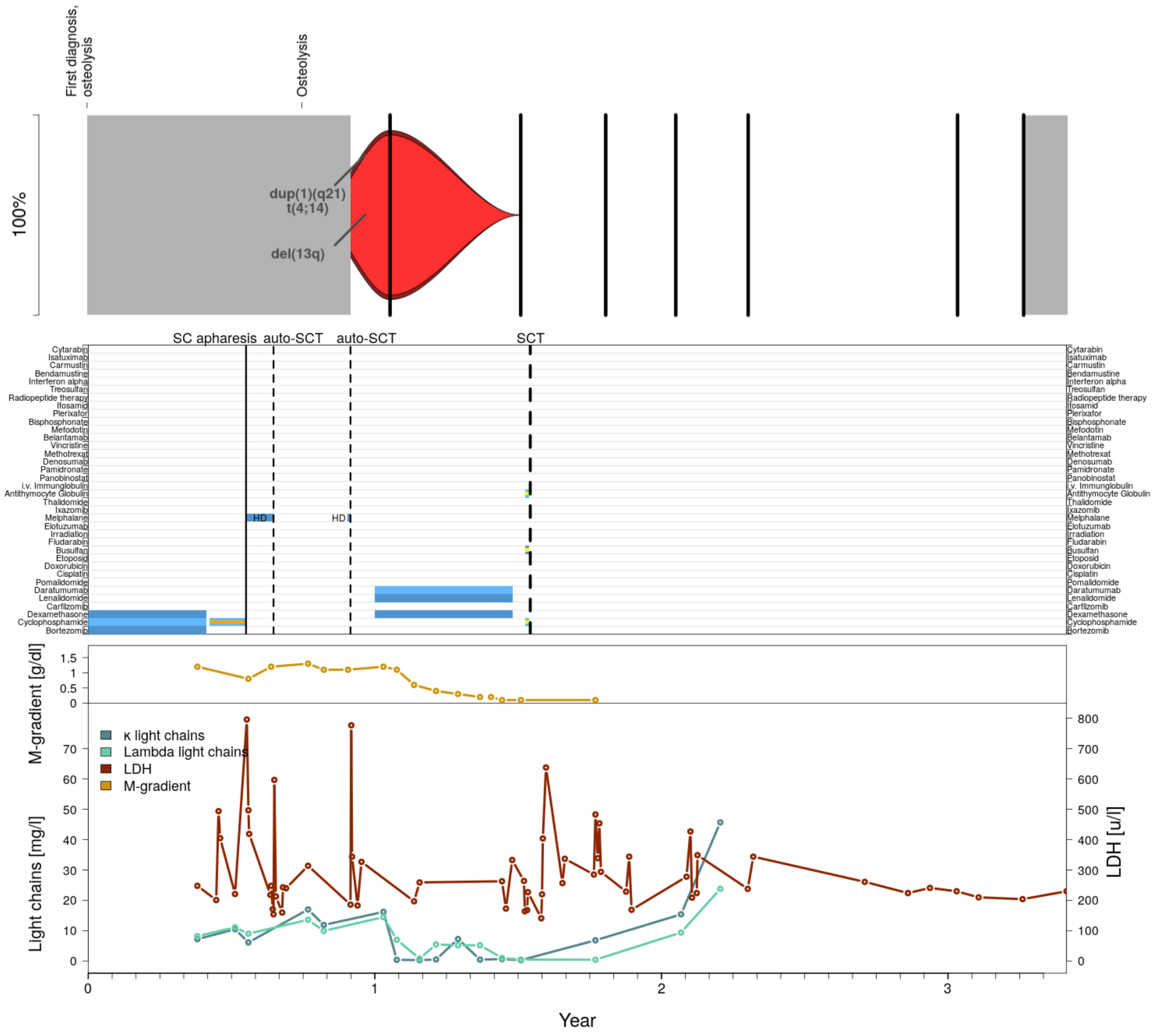

Supplementary Figure 12: Clonal evolution, applied therapies and development of laboratory parameters of patient UPN08. Vertical black lines in the clonal evolution plot indicate the time points of aberration analysis; auto-SCT: autologous stem cell transplantation, HD – high dose, SC – stem cell, SCT – stem cell transplantation.

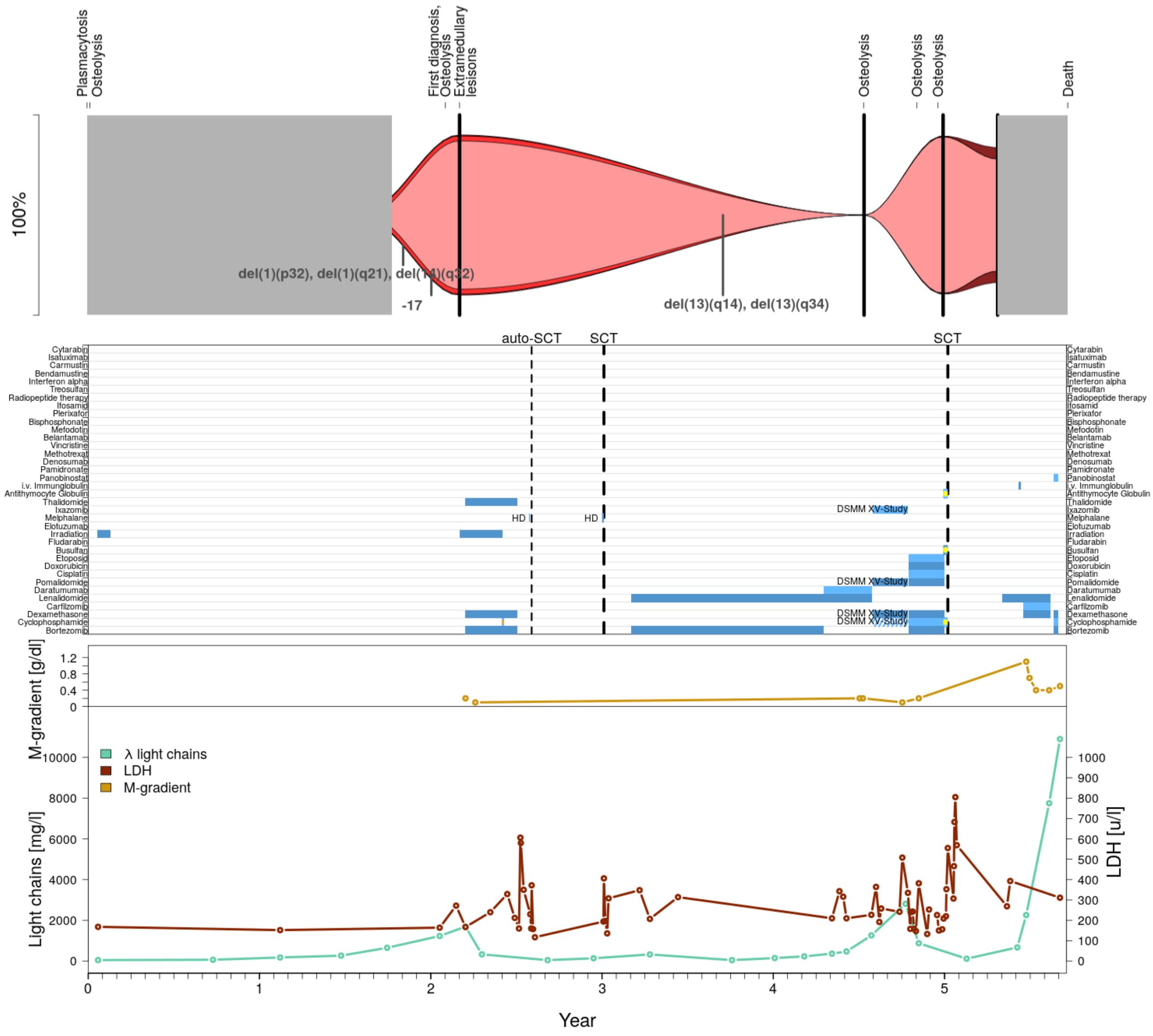

Supplementary Figure 13: Clonal evolution, applied therapies and development of laboratory parameters of patient UPN09. Vertical black lines in the clonal evolution plot indicate the time points of aberration analysis; auto-SCT – autologous stem cell transplantation, HD – high dose, SC – stem cell, SCT – stem cell transplantation.

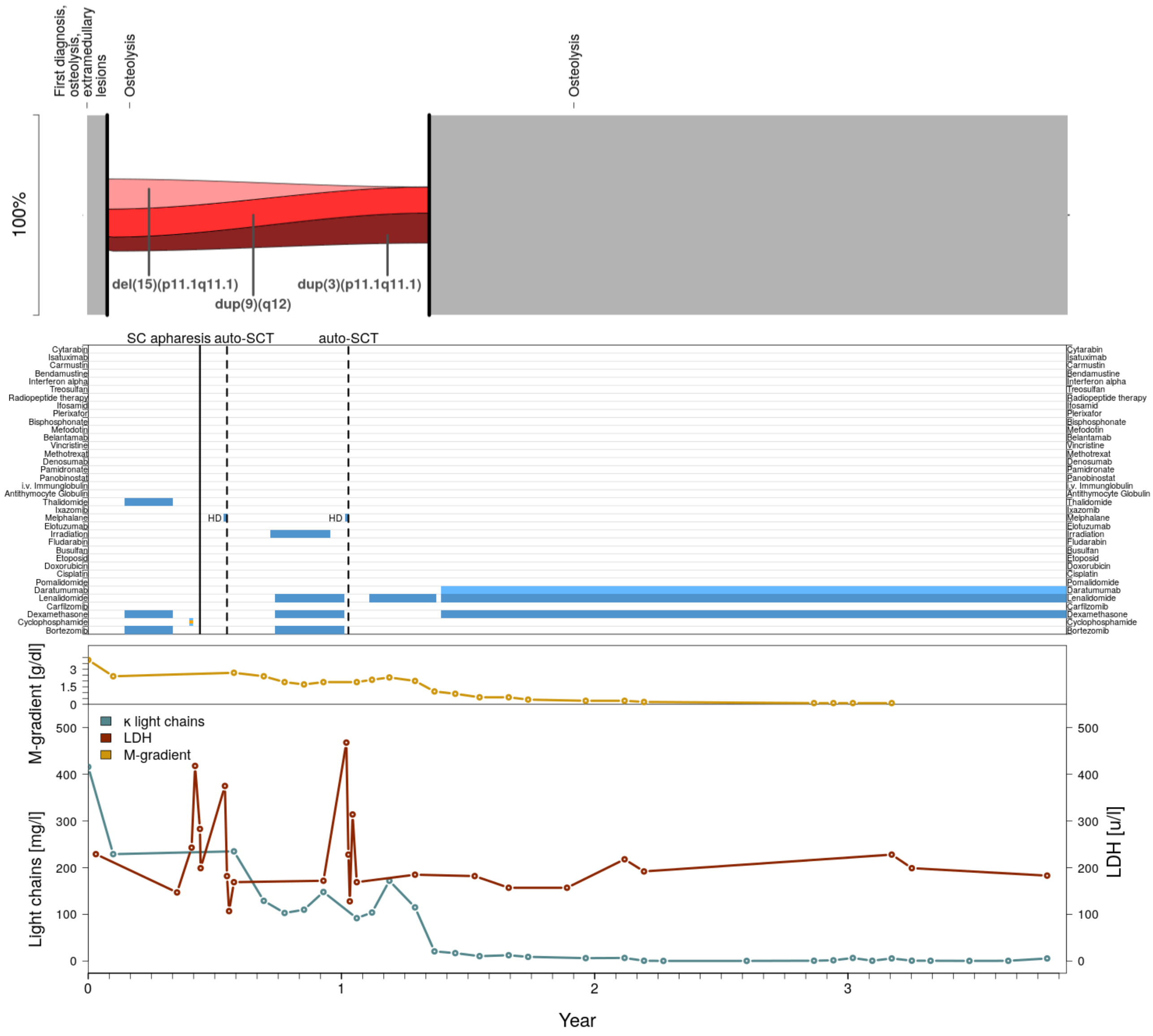

Supplementary Figure 14: Clonal evolution, applied therapies and development of laboratory parameters of patient UPN10. Vertical black lines in the clonal evolution plot indicate the time points of aberration analysis; auto-SCT – autologous stem cell transplantation, HD – high dose, SC – stem cell.

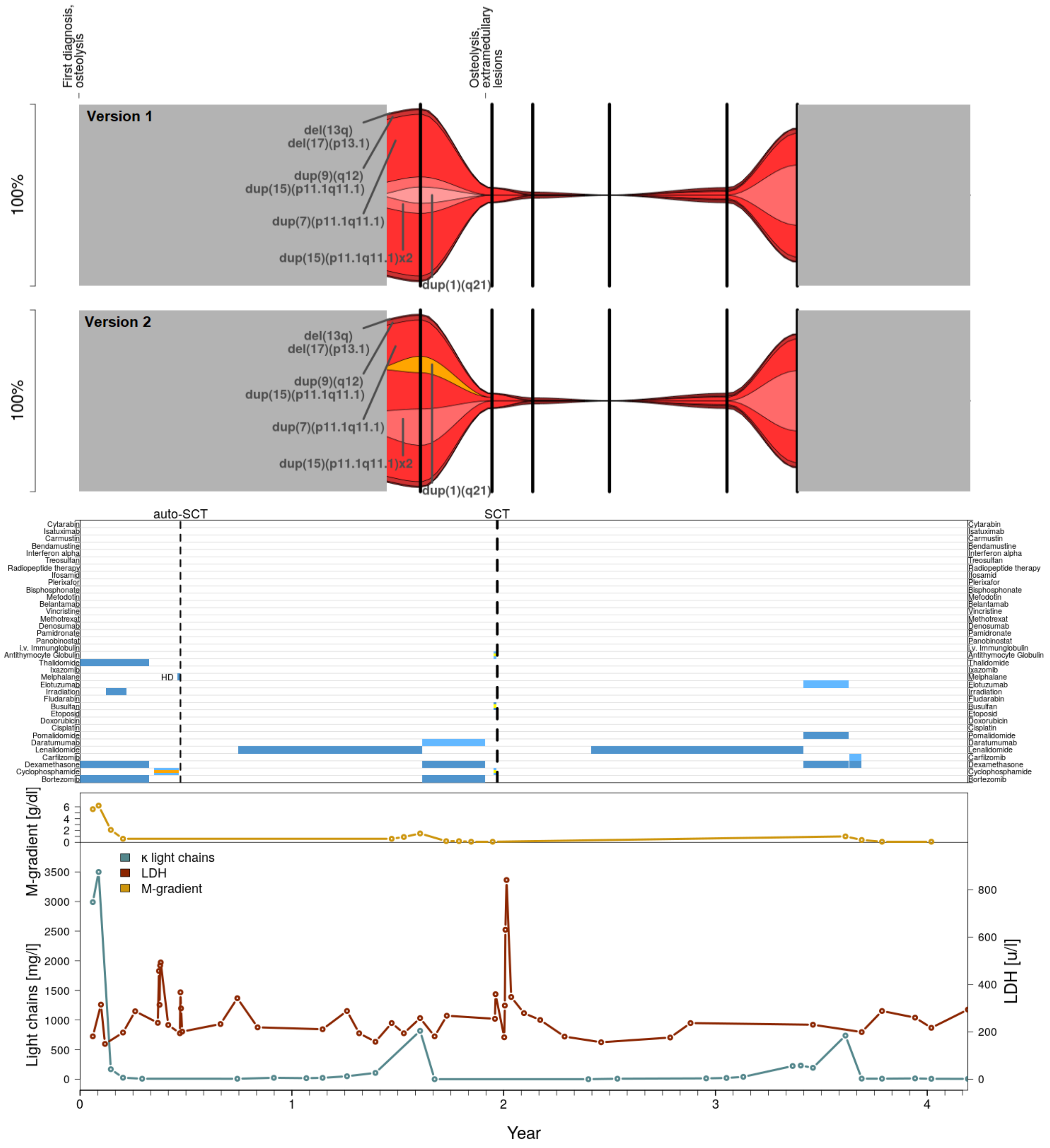

Supplementary Figure 15: Clonal evolution (2 possible versions), applied therapies and development of laboratory parameters of patient UPN11. Vertical black lines in the clonal evolution plot indicate the time points of aberration analysis; auto-SCT – autologous stem cell transplantation, HD – high dose, SCT – stem cell transplantation.

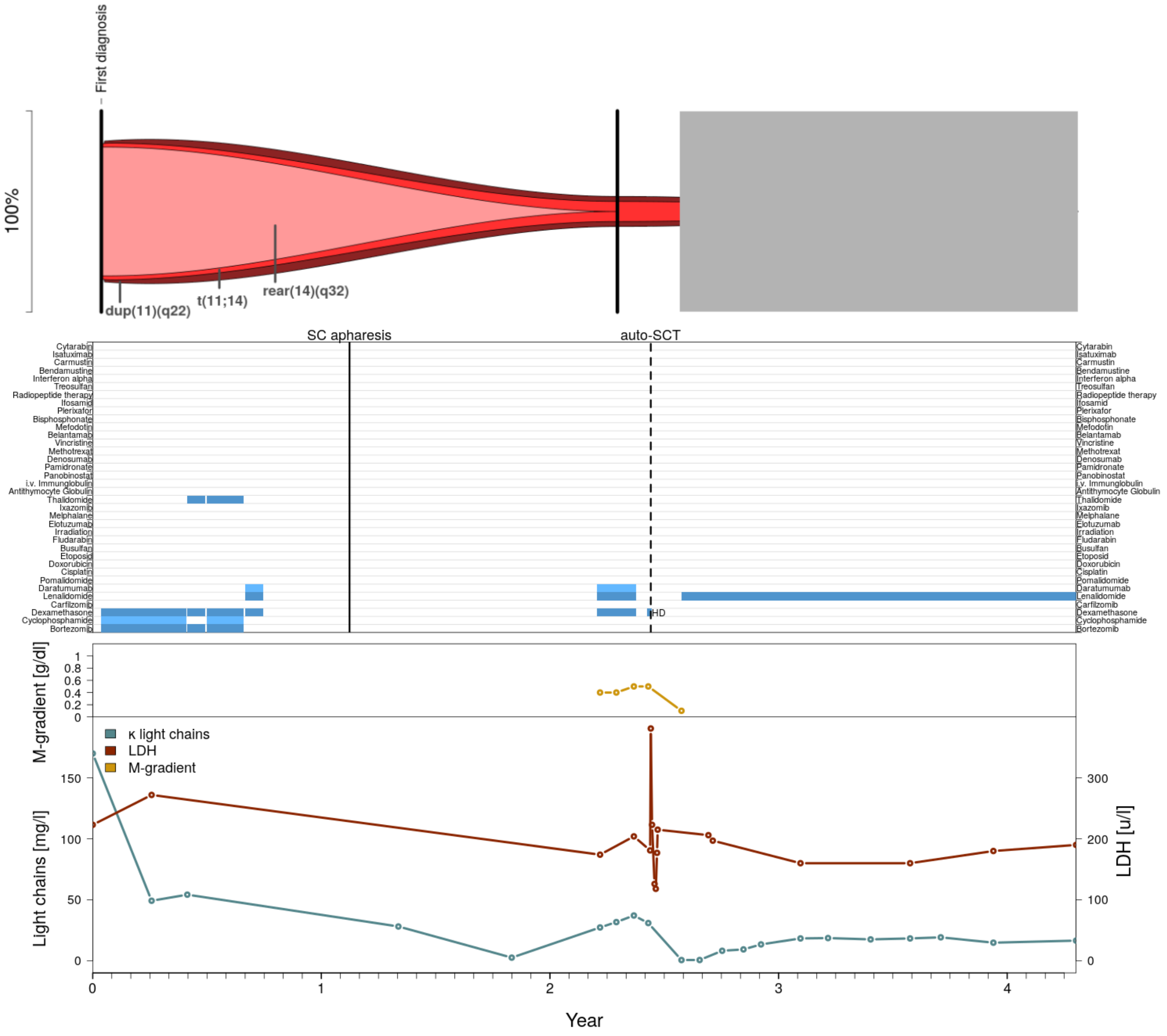

Supplementary Figure 16: Clonal evolution, applied therapies and development of laboratory parameters of patient UPN12. Vertical black lines in the clonal evolution plot indicate the time points of aberration analysis; auto-SCT – autologous stem cell transplantation, HD – high dose, SC – stem cell.

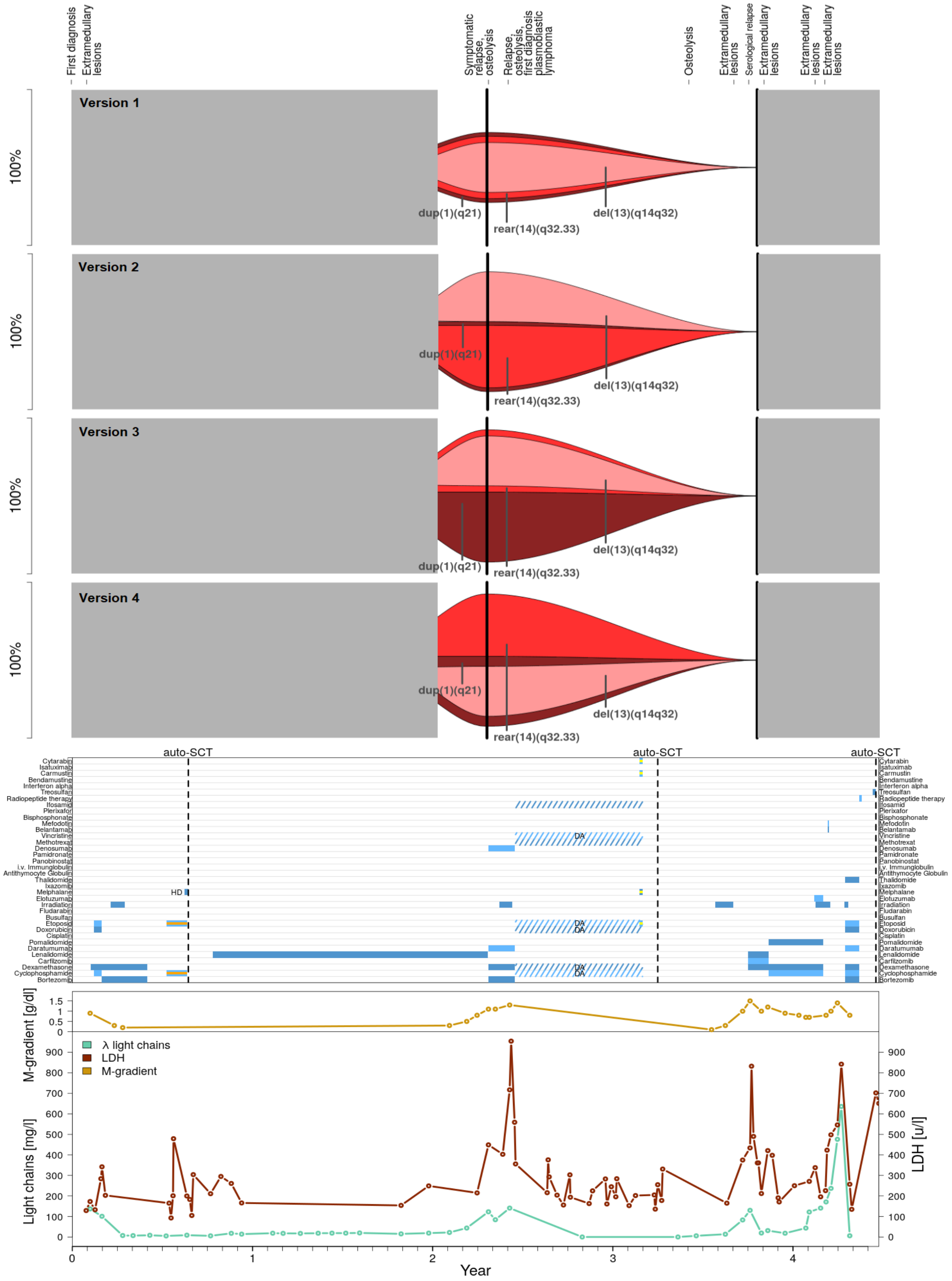

Supplementary Figure 17: Clonal evolution (4 possible versions), applied therapies and development of laboratory parameters of patient UPN13. Vertical black lines in the clonal evolution plot indicate the time points of aberration analysis; auto-SCT – autologous stem cell transplantation, DA – dose adjusted, HD – high dose, SC – stem cell.

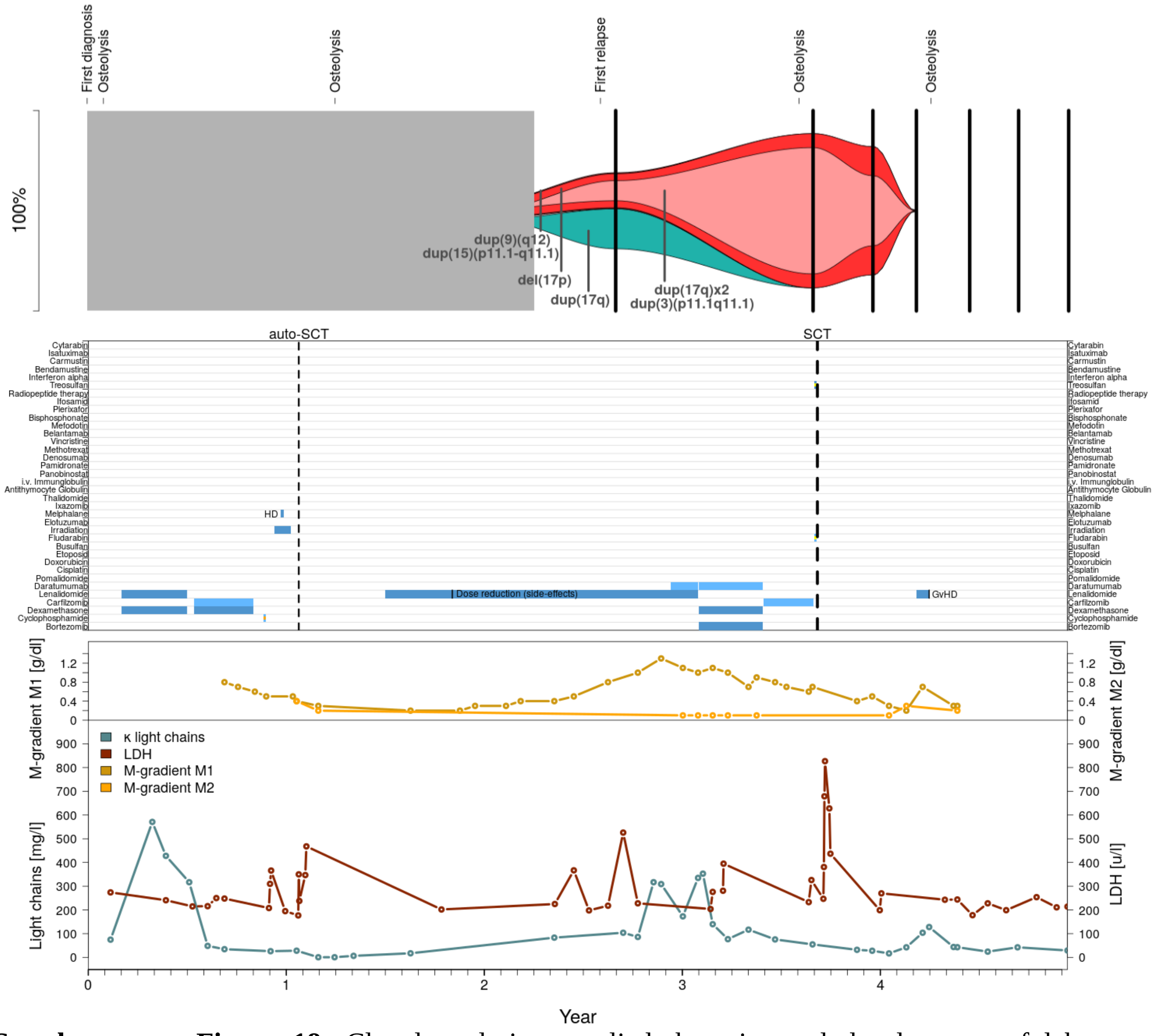

Supplementary Figure 18: Clonal evolution, applied therapies and development of laboratory parameters of patient UPN14. Vertical black lines in the clonal evolution plot indicate the time points of aberration analysis; auto-SCT – autologous stem cell transplantation, GvHD – graft-versus-host disease, HD – high dose, SCT – stem cell transplantation.

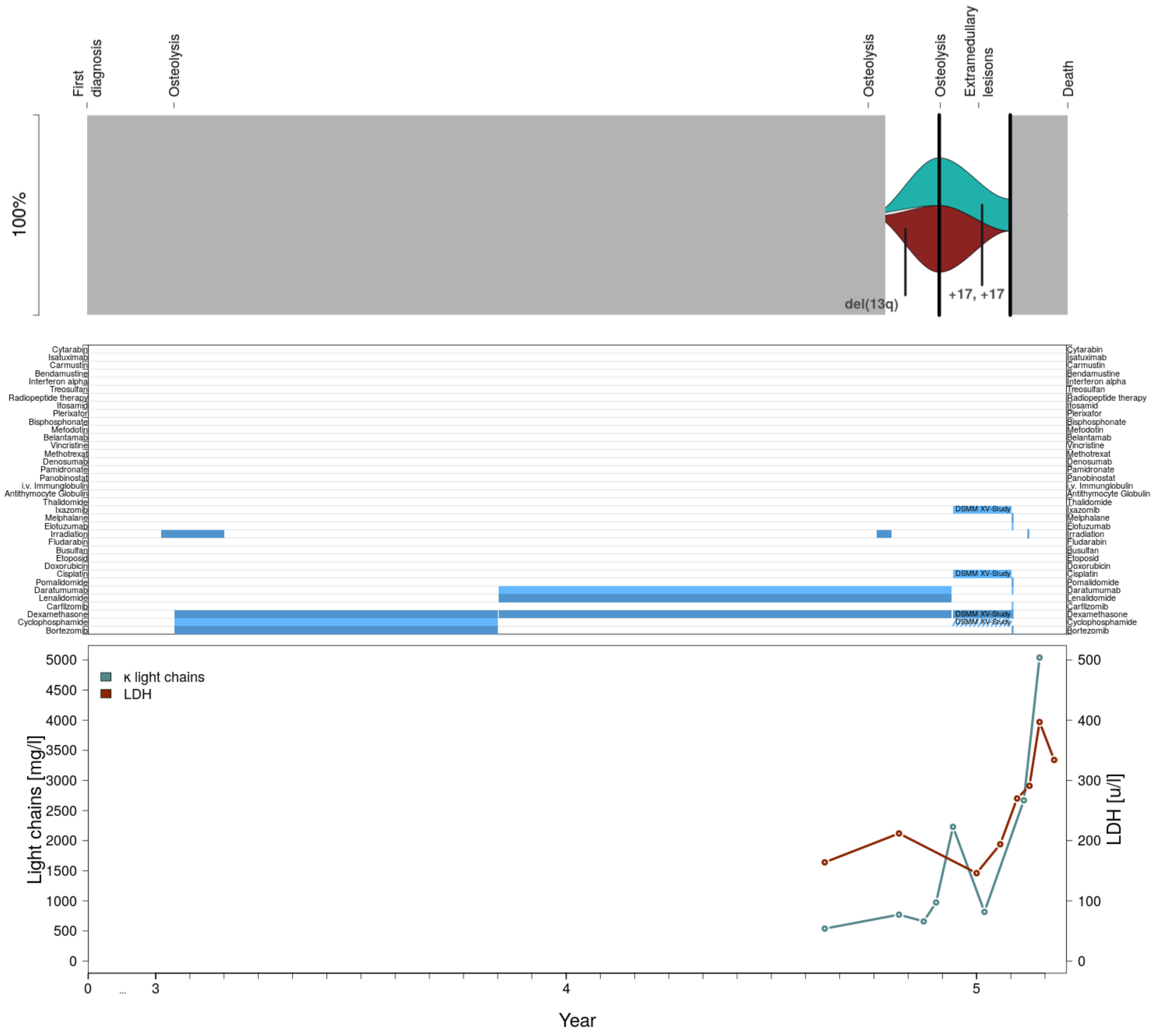

Supplementary Figure 19: Clonal evolution, applied therapies and development of laboratory parameters of patient UPN15. Vertical black lines in the clonal evolution plot indicate the time points of aberration analysis.

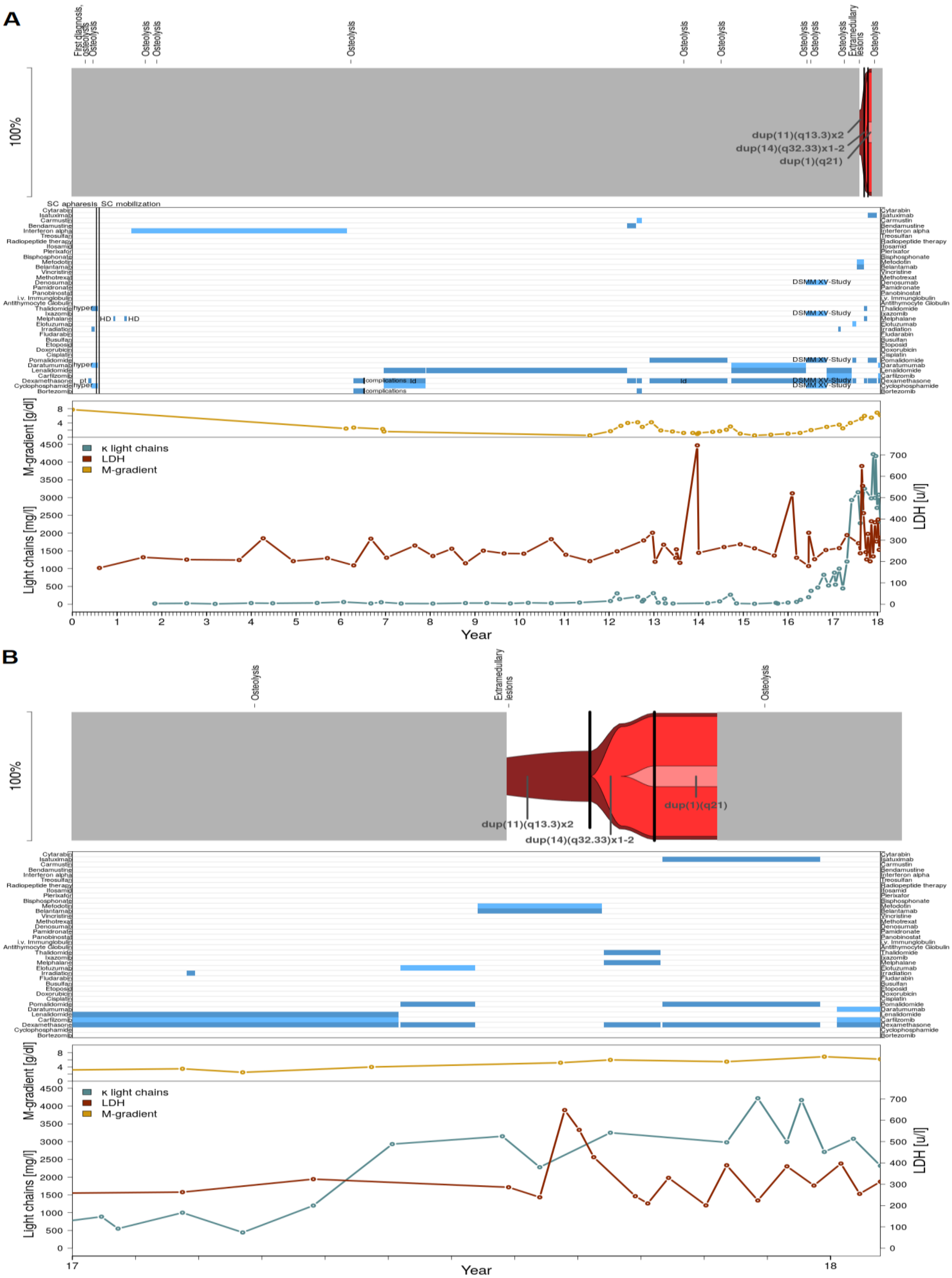

Supplementary Figure 20: Clonal evolution, applied therapies and development of laboratory parameters of patient UPN16. Vertical black lines in the clonal evolution plot indicate the time points of aberration analysis; HD – high dose, ld – low dose, pt – pulse therapy, SC – stem cell. A) Full time of follow-up. B) Starting 17 years after first diagnosis.

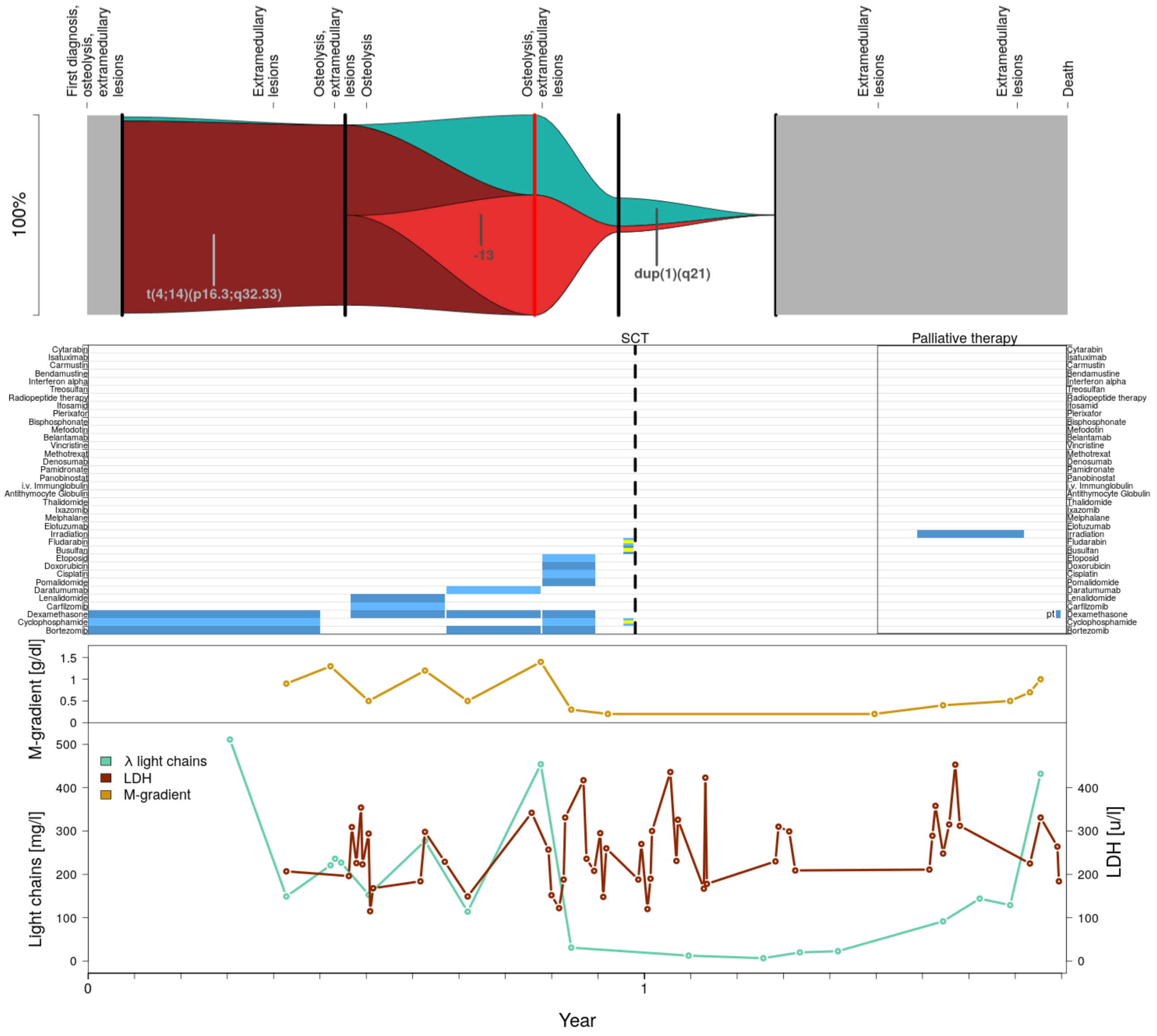

Supplementary Figure 21: Clonal evolution, applied therapies and development of laboratory parameters of patient UPN17. Vertical black lines in the clonal evolution plot indicate the time points of aberration analysis, vertical red line indicates the time point of aberration analysis at which a new clone was detected; SCT – stem cell transplantation.

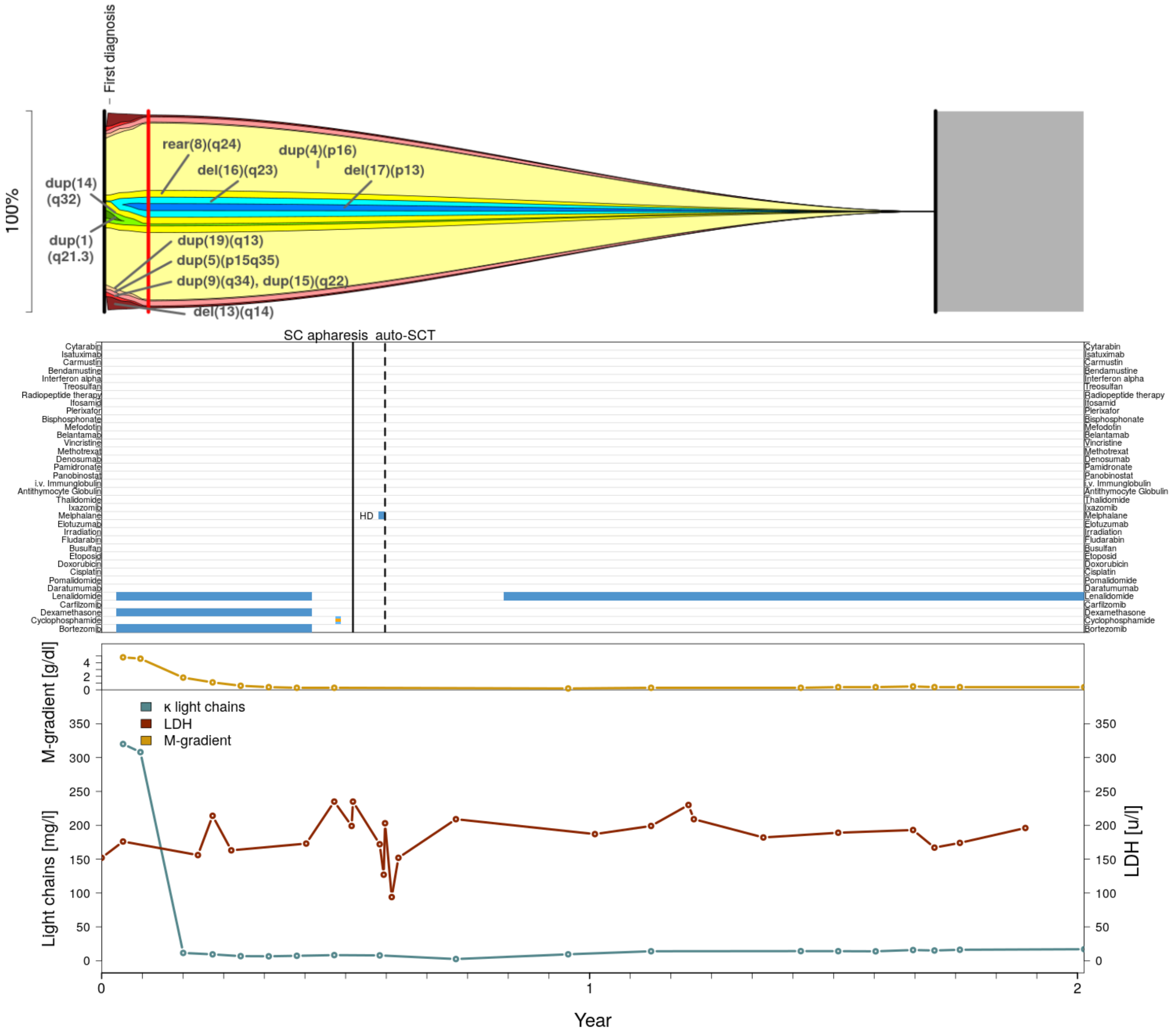

Supplementary Figure 22: Clonal evolution, applied therapies and development of laboratory parameters of patient UPN18. Vertical black lines in the clonal evolution plot indicate the time points of aberration analysis, vertical red line indicates the time point of aberration analysis at which a new clone was detected; auto-SCT – autologous stem cell transplantation, HD – high dose, SC – stem cell.

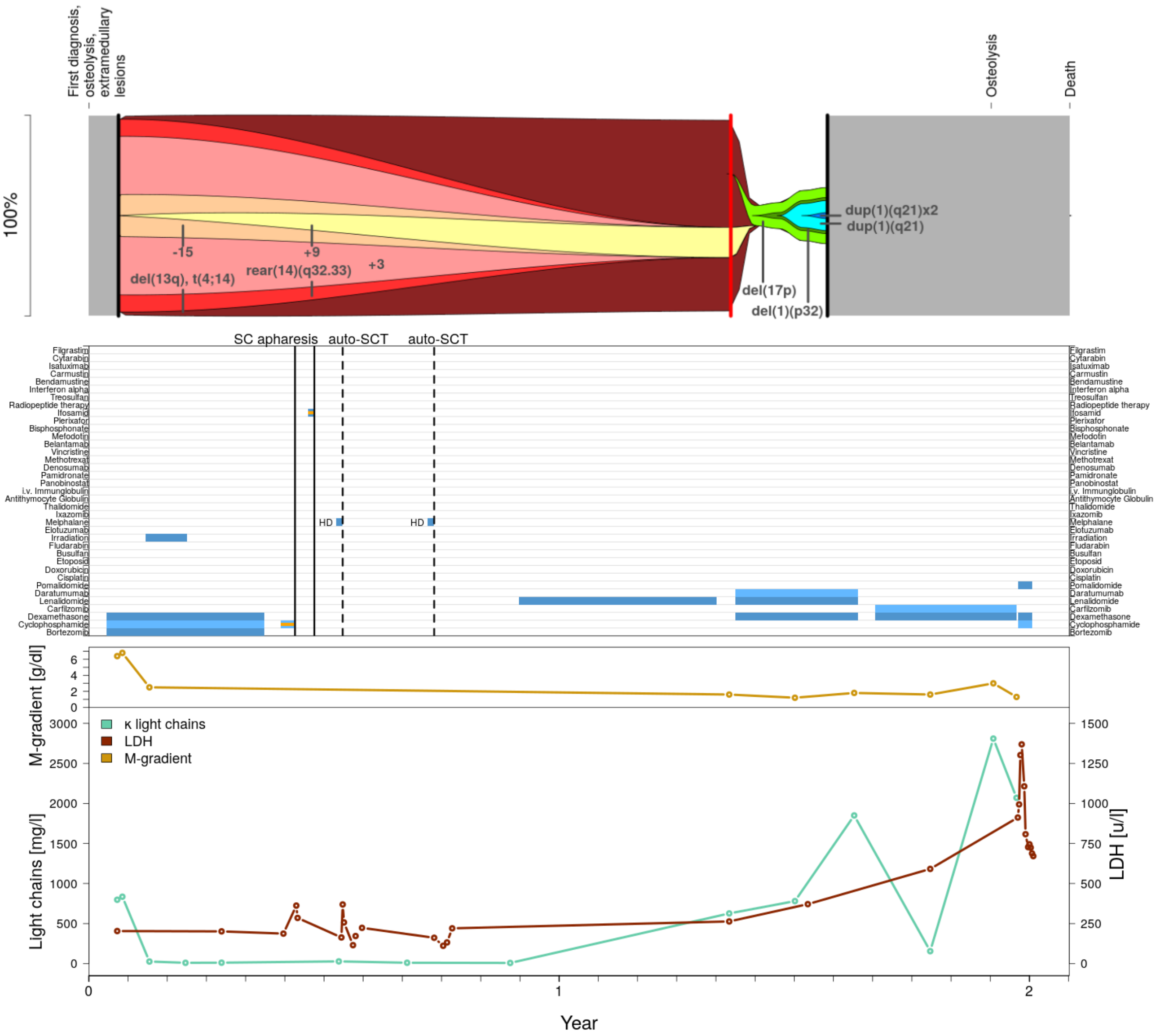

Supplementary Figure 23: Clonal evolution, applied therapies and development of laboratory parameters of patient UPN19. Vertical black lines in the clonal evolution plot indicate the time points of aberration analysis, vertical red line indicates the time point of aberration analysis at which a new clone was detected; auto-SCT – autologous stem cell transplantation, HD – high dose, SC – stem cell.

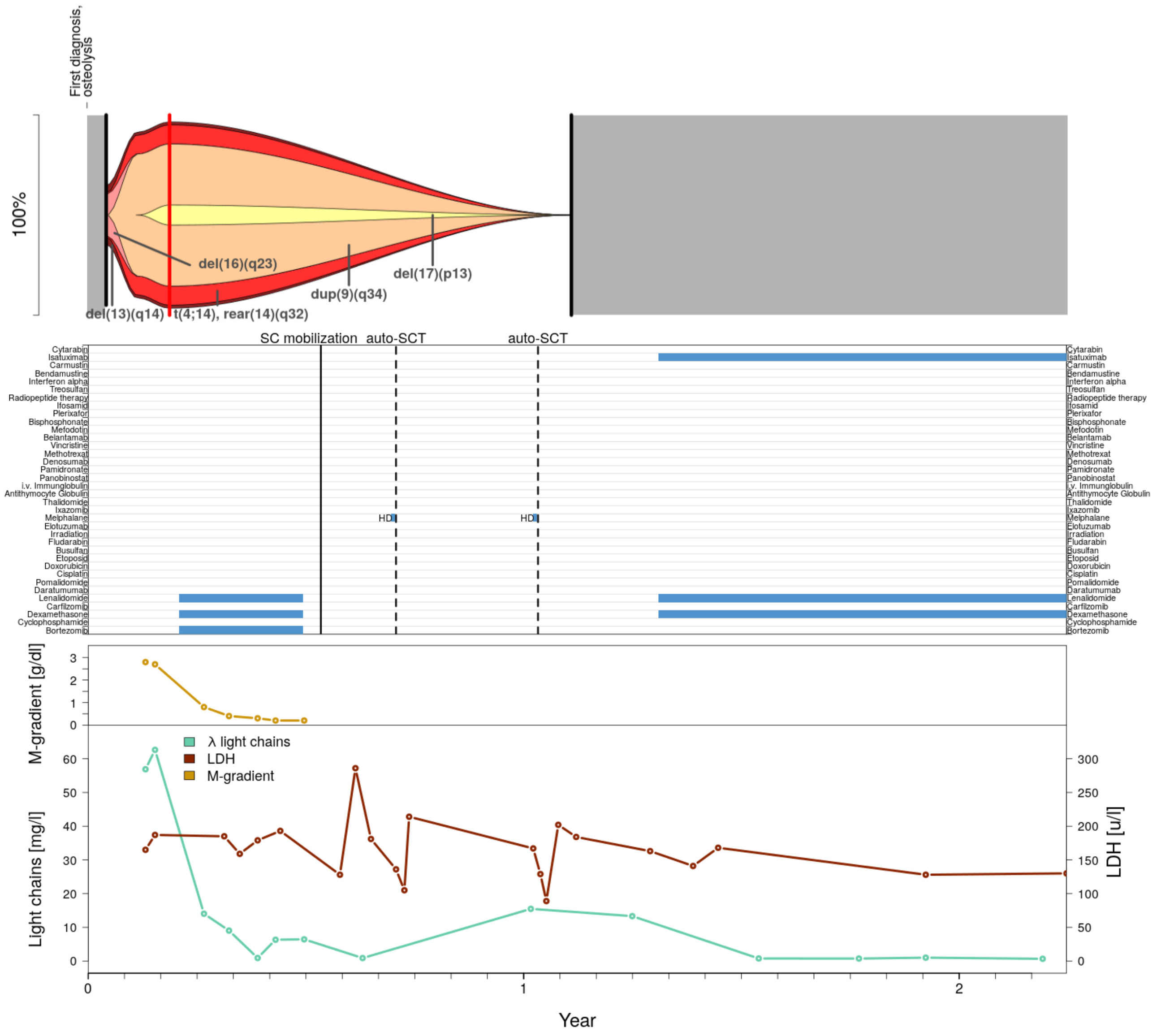

Supplementary Figure 24: Clonal evolution, applied therapies and development of laboratory parameters of patient UPN20. Vertical black lines in the clonal evolution plot indicate the time points of aberration analysis, vertical red line indicates the time point of aberration analysis at which a new clone was detected; auto-SCT – autologous stem cell transplantation, HD – high dose, SC – stem cell.

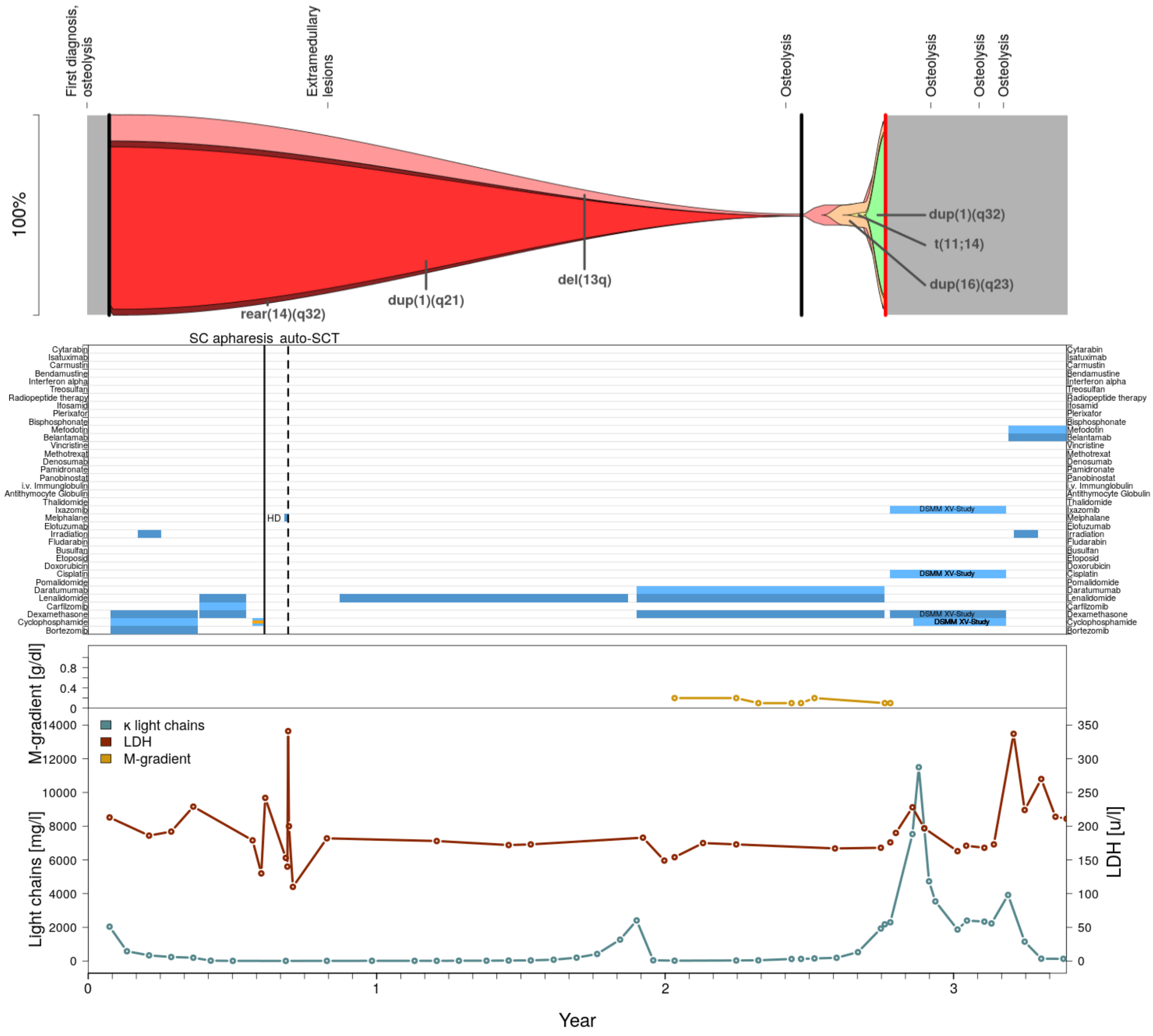

Supplementary Figure 25: Clonal evolution, applied therapies and development of laboratory parameters of patient UPN21. Vertical black lines in the clonal evolution plot indicate the time points of aberration analysis, vertical red line indicates the time point of aberration analysis at which a new clone was detected; auto-SCT – autologous stem cell transplantation, HD – high dose, SC – stem cell.

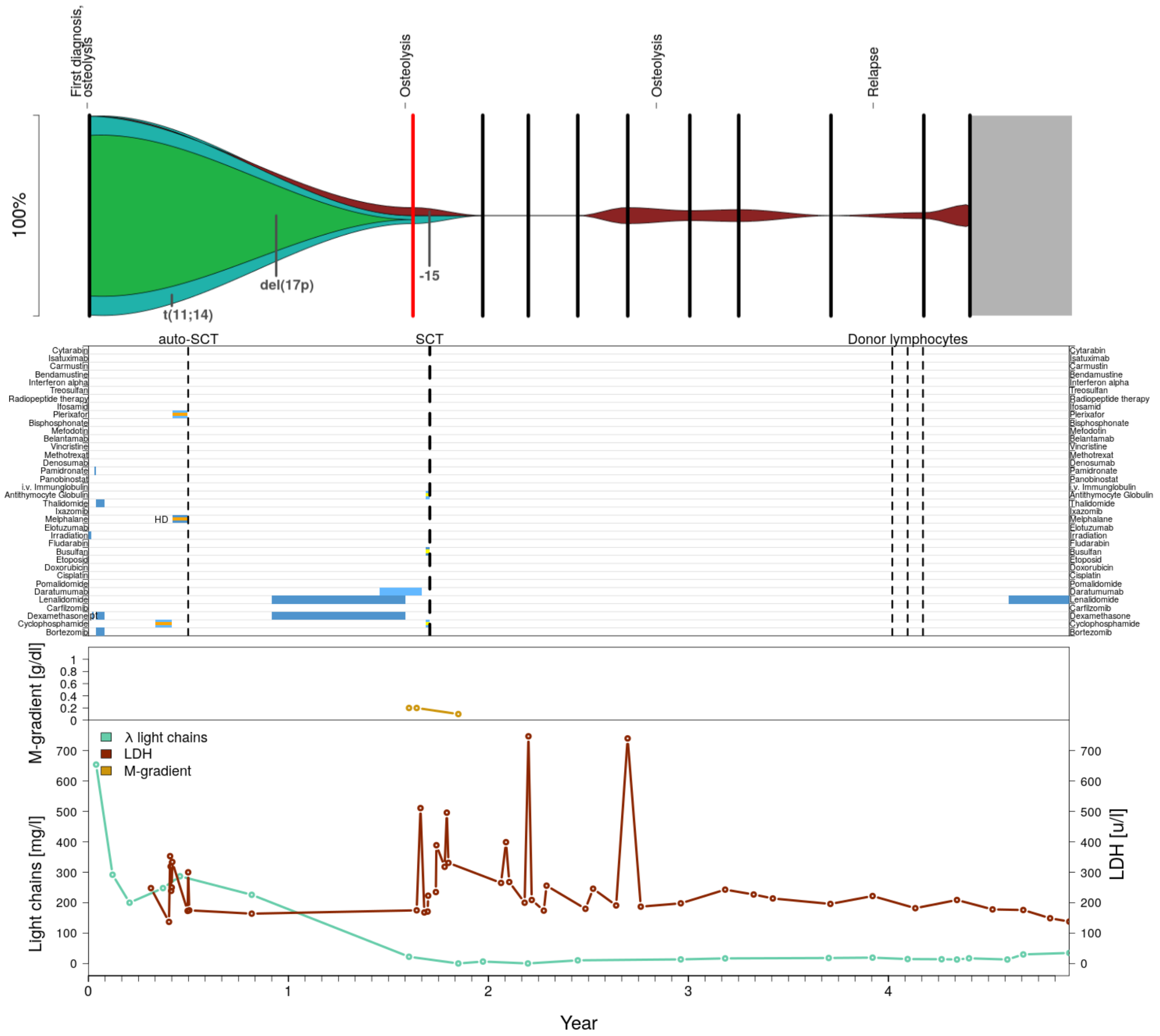

Supplementary Figure 26: Clonal evolution, applied therapies and development of laboratory parameters of patient UPN22. Vertical black lines in the clonal evolution plot indicate the time points of aberration analysis, vertical red line indicates the time point of aberration analysis at which a new clone was detected; auto-SCT – autologous stem cell transplantation, HD – high dose, SCT – stem cell transplantation.

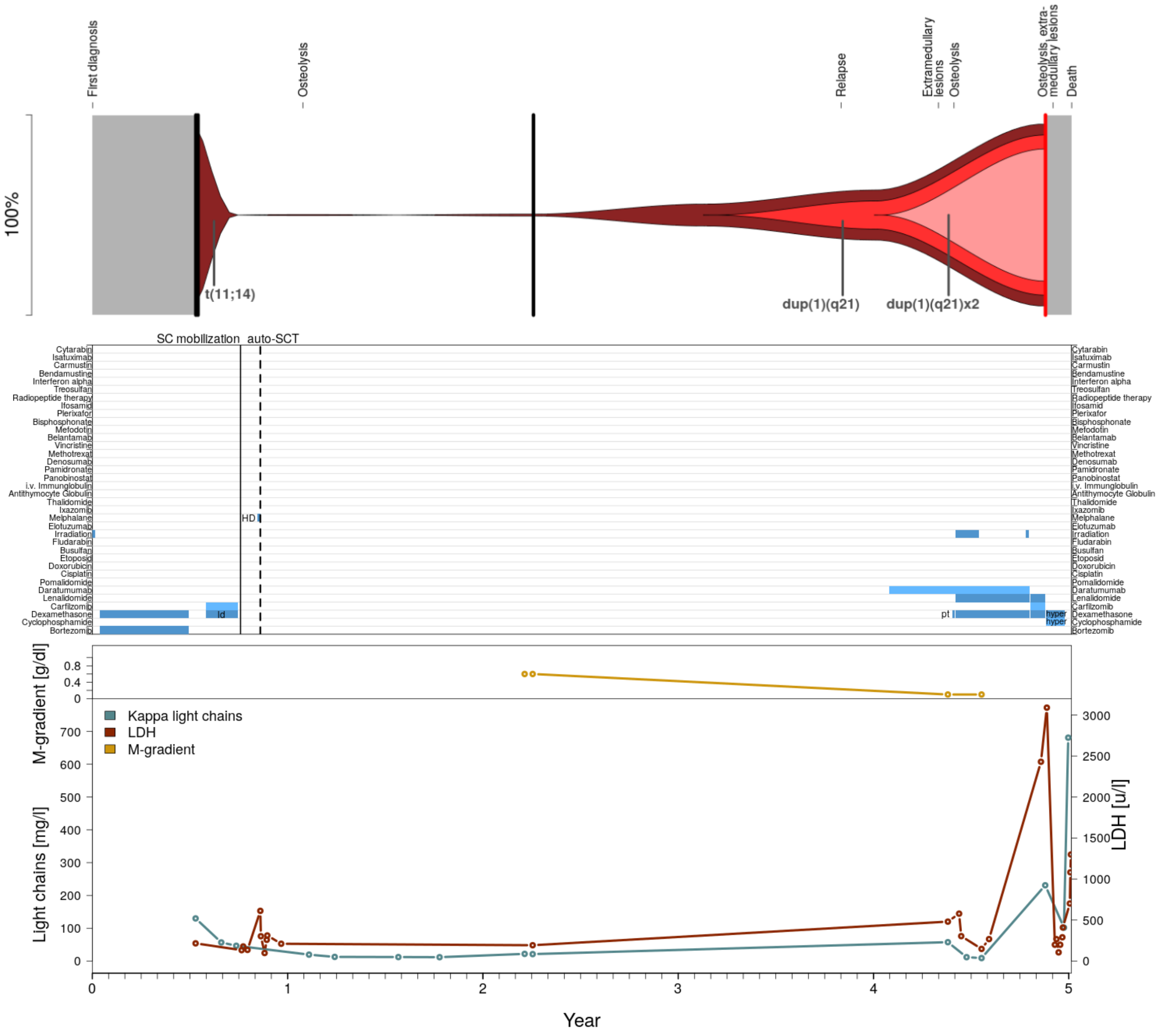

Supplementary Figure 27: Clonal evolution, applied therapies and development of laboratory parameters of patient UPN23. Vertical black lines in the clonal evolution plot indicate the time points of aberration analysis, vertical red line indicates the time point of aberration analysis at which a new clone was detected; auto-SCT – autologous stem cell transplantation, HD – high dose, ld – low dose, pt – pulse therapy SC – stem cell.

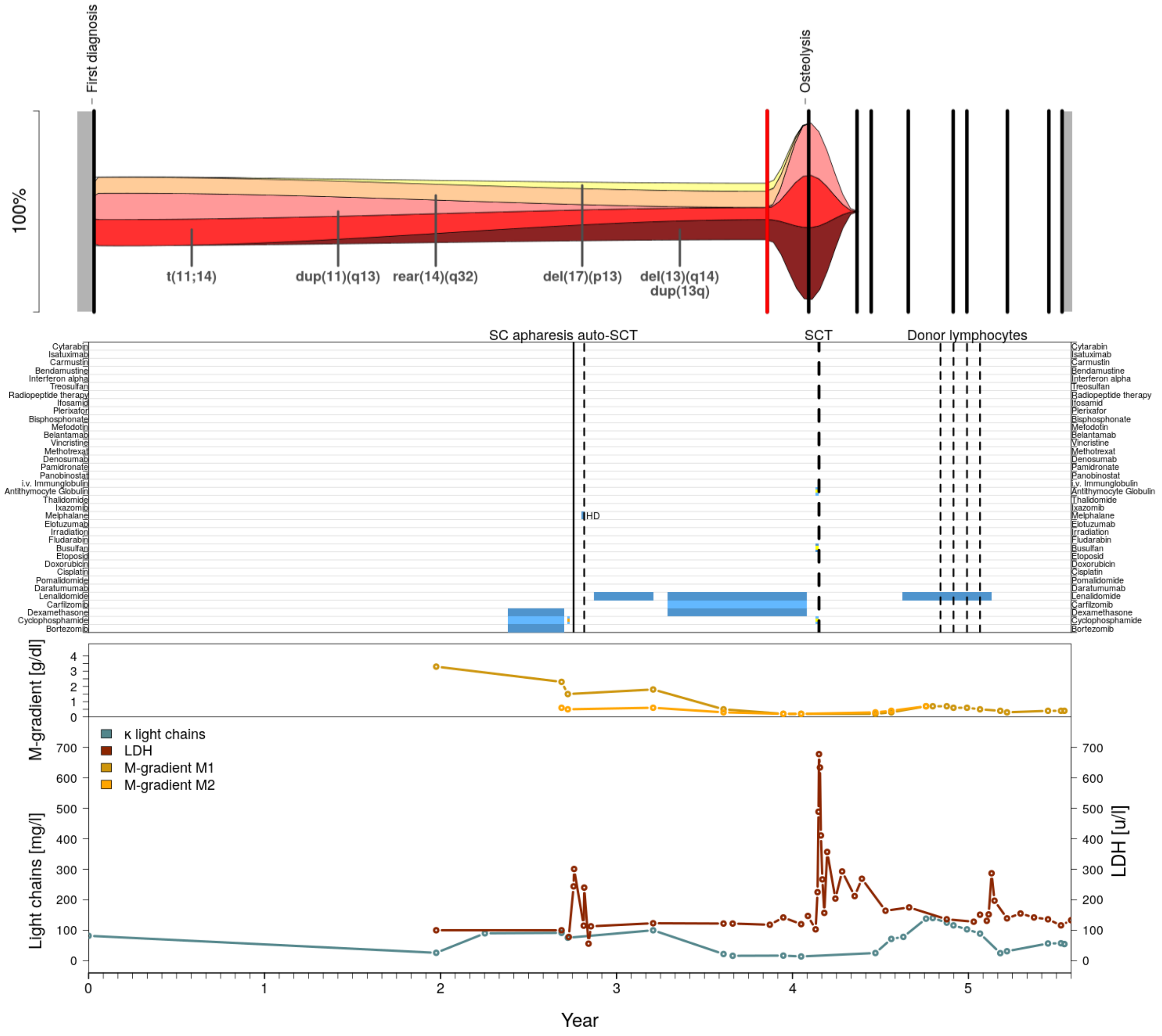

Supplementary Figure 28: Clonal evolution, applied therapies and development of laboratory parameters of patient UPN24. Vertical black lines in the clonal evolution plot indicate the time points of aberration analysis, vertical red line indicates the time point of aberration analysis at which a new clone was detected; auto-SCT – autologous stem cell transplantation, HD – high dose, SC – stem cell, SCT – stem cell transplantation.

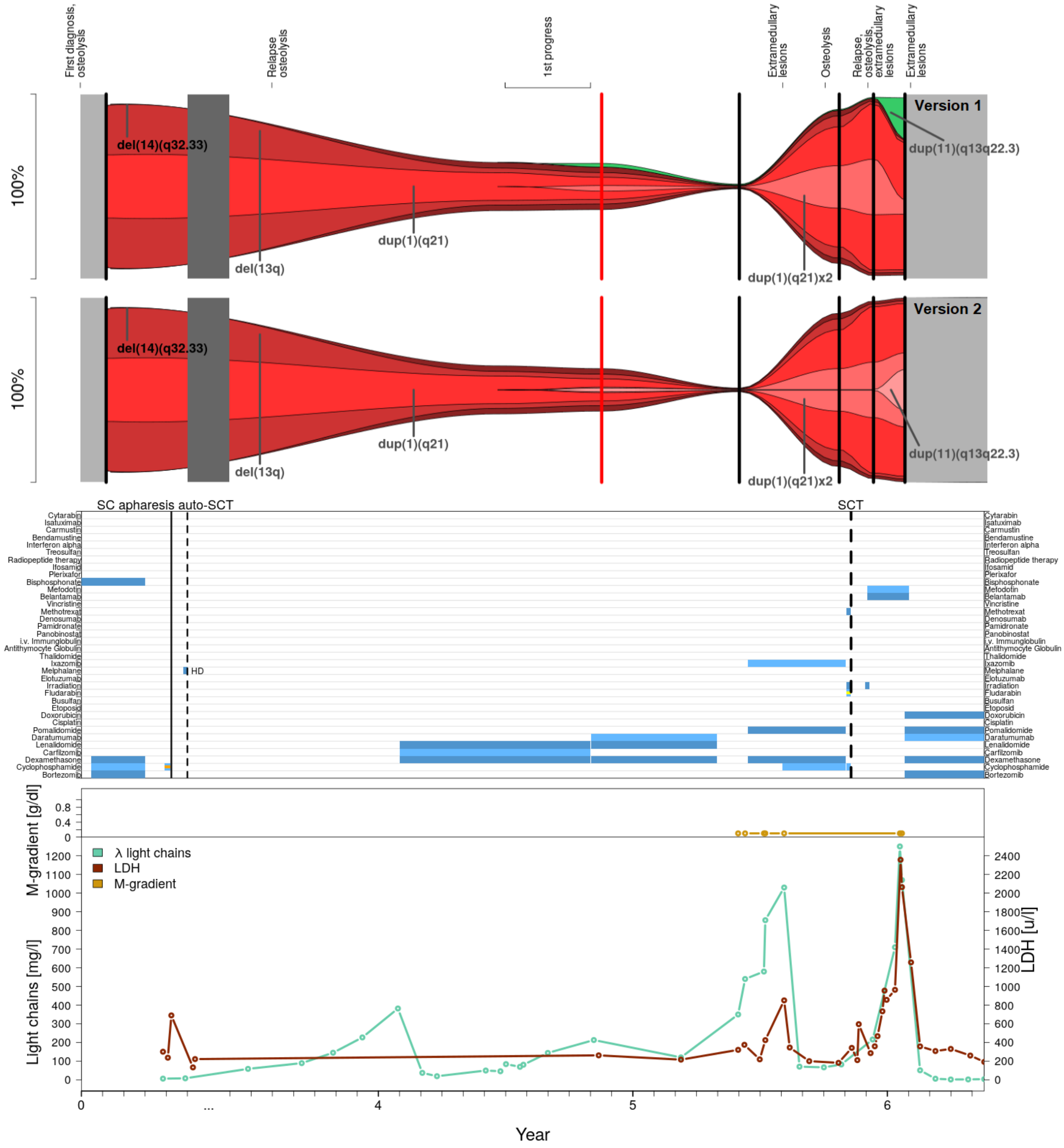

Supplementary Figure 29: Clonal evolution (2 possible versions), applied therapies and development of laboratory parameters of patient UPN25. Vertical black lines in the clonal evolution plot indicate the time points of aberration analysis, vertical red line indicates the time point of aberration analysis at which a new clone was detected; auto-SCT – autologous stem cell transplantation, HD – high dose, SC – stem cell, SCT – stem cell transplantation.
